# Middle Jurassic stem hynobiids from China shed light on the evolution of basal salamanders

**DOI:** 10.1016/j.isci.2021.102744

**Published:** 2021-06-17

**Authors:** Jia Jia, Jason S. Anderson, Ke-Qin Gao

**Affiliations:** 1 School of Earth and Space Sciences, Peking University, 5 Yiheyuan Road, Beijing 100871, China; 2 State Key Laboratory of Palaeobiology and Stratigraphy (Nanjing Institute of Geology and Palaeontology, CAS), 39 East Beijing Road, Nanjing, Jiangsu Province 210008, China; 3 Department of Comparative Biology and Experimental Medicine, University of Calgary, 3330 Hospital Drive, Calgary, AB T2N 4N1, Canada

**Keywords:** Biological Sciences, Zoology, Evolutionary Biology, Phylogeny

## Abstract

The Hynobiidae are an early-diverging clade of crown-group salamanders (urodeles) with an important bearing on the evolution of urodeles. Paleobiology and early-branching patterns of the Hynobiidae remain unclear owing to a poorly documented fossil record. We reported a newly referred specimen to the stem hynobiid, originally named as “*Liaoxitriton daohugouensis,*” but here as *Neimengtriton daohugouensis* comb. nov., and predates the previously estimated origination time of Hynobiidae for at least 8 Myr. We interpret *N. daohugouensis* as semiaquatic at the adult stage, a previously unknown paleoecological preference among Mesozoic salamanders. Phenotypic variations of *N. daohugouensis* enlighten an unrecognized association between caudosacral vertebrae and fertilization modes in the early evolution of urodeles. Our cladistic analyses based on morphological characters not only recognize several stem hynobiids and establish Panhynobia *nomen cladinovum* for the total-group hynobiids but also shed light on the sequential evolution of morphological features in this primitive urodele clade.

## Highlights

• The Middle Jurassic “*Liaoxitriton*” *daohugouensis* is not congenic with *Liaoxitriton zhongjiani*
• *Neimengtriton daohugouensis* comb. nov. is a semiaquatic stem hynobiid salamander
• Sequential morphological evolutionary patterns were revealed for Cryptobranchoidea
• Variations in caudosacral numbers are associated with fertilization mode evolution

## Graphical Abstract

**Figure:**
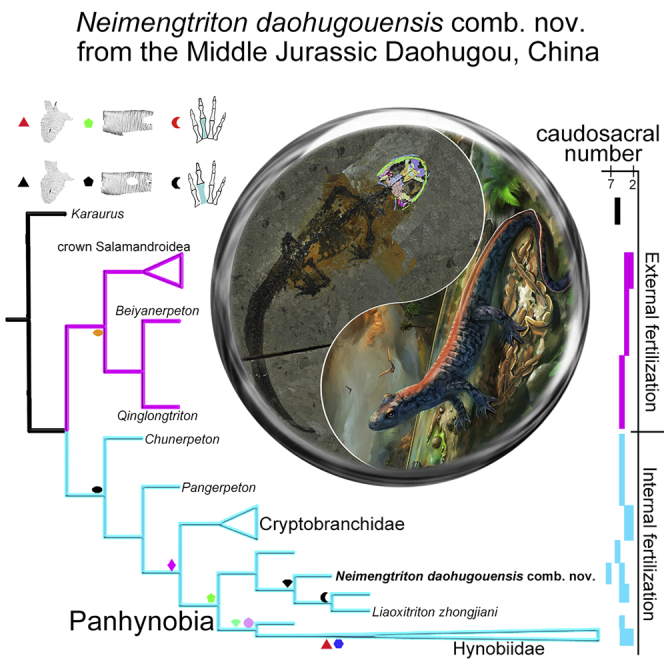

## Introduction

Hynobiidae, commonly known as Asiatic salamanders, encompass 83 or 85 extant species in nine or ten genera (validity of *Protohynobius* as pending) that live primarily in Asia, with a single species (*Salamandrella keyserlingii*) extending into the European part of Russia (e.g., Duellman and Trueb, 1986; Blackburn and Wake, 2011; Vitt and Caldwell, 2014; Fei and Ye, 2016; AmphibiaWeb, 2021; Frost, 2021). Extant hynobiids are small to medium sized (70–270 mm in total length) and typically undergo metamorphosis, the life history process during which all larval structures (e.g., external gills) are lost by resorption to transform larvae into adults (e.g., Dunn, 1923; Noble, 1931; Duellman and Trueb, 1986; Larson et al., 2003; Fei and Ye, 2016; but see Jiang et al., 2018). Postmetamorphosed hynobiids can be terrestrial, aquatic, or semiaquatic, and all have external fertilization breeding by spawning in water (e.g., Regal, 1966; Kuzmin and Thiesmeier, 2001; Poyarkov et al., 2012; Fei and Ye, 2016; see Discussion).

Phylogenetically, the Hynobiidae are united with Cryptobranchidae as sister groups within the suborder Cryptobranchoidea (e.g., Estes, 1981; Duellman and Trueb, 1986; Larson et al., 2003; Zhang et al., 2006; Zhang and Wake, 2009; Pyron and Wiens, 2011; Weisrock et al., 2013; Chen et al., 2015), which has long been regarded as a primitive clade among the crown salamanders, or Urodela (Dunn, 1922), because cryptobranchoids retained several plesiomorphic features of urodeles, including the retention of an angular bone in the mandible and breeding by external fertilization (e.g., Estes, 1981; Duellman and Trueb, 1986; Jia and Gao, 2016a; 2019). Interestingly, some if not all hynobiids have several atavistic features (e.g., two centralia in wrist/ankle) either as standard patterns or intraspecific variations that are lost in derived urodeles but are present in the hypothesized temnospondyl ancestors of modern amphibians (e.g., Schmalhausen, 1968; Shubin and Wake, 2003; Boisvert, 2009; Jiang et al., 2018; Jia et al., 2019).

The evolutionary history of the Hynobiidae is poorly known because fossil records of this family are one of the least well documented among urodele clades (Estes, 1981; Gao and Shubin, 2012). Cenozoic fossils of hynobiids are known only by isolated bones from the Miocene through Holocene in Asia and Eastern Europe that are either affiliated with two extant genera, *Salamandrella* and *Ranodon* (e.g., Averianov and Tjutkova, 1995; Ratnikov, 2010; Vasilyan et al., 2013; Syromyatnikova, 2014), and the extinct genus *Parahynobius* (Venczel, 1999a; 1999b; Venczel and Hír, 2013; but see Jia and Gao, 2016a), or were referred to as Hynobiidae indet (e.g., Ratnikov, 2010; Vasilyan et al., 2017). In the Mesozoic, the genera *Iridotriton* (Tithonian) from the USA and *Kiyatriton* (Aptian-Albian and Bathonian) from Siberian Russia are known by a disarticulated skeleton and isolated bones, respectively (Evans et al., 2005; Skutschas, 2014, 2016). Although more or less hynobiid-like (e.g., Gao and Shubin, 2012; Skutschas, 2014), both of them are at best to be treated as members of the Cryptobranchoidea (Skutschas, 2016; Jia and Gao, 2016b, 2019; Rong, 2018). The most promising stem hynobiids are eight species in seven genera that belong to the Early Cretaceous Jehol Biota (*Laccotriton*, *Liaoxitriton*, *Nuominerpeton*, *Regalerpeton*, and *Sinerpeton*) and the Middle–Late Jurassic Yanliao Biota (*Liaoxitriton*, *Linglongtriton*, and *Pangerpeton*; Jia and Gao, 2019). All these species are known from well-articulated specimens superbly preserved in lacustrine deposits in Inner Mongolia, Liaoning, and Hebei provinces, China (Gao et al., 2013; Jia and Gao, 2016a, 2019).

Most of the Chinese Mesozoic salamander genera are monotypic except *Liaoxitriton*, which contains two nominal species, implying that the genus is the only vertebrate component occurred in both the Jehol and Yanliao biotas (Zhou and Wang, 2017). The type species *Liaoxitriton zhongjiani* Dong and Wang (1998) is known by more than 30 specimens from the Lower Cretaceous Yixian Formation in western Liaoning (see Gao et al., 2013), whereas the other nominal species “*Liaoxitriton daohugouensis*” is erected on three specimens from the Daohugou beds of Inner Mongolia (Wang, 2004a; Sullivan et al., 2014). Conversely, our study of a fourth specimen from the type locality showed that “*L*. *daohugouensis*” cannot be properly classified as congeneric with the type species, but instead, it ought to be assigned to a genus of its own on the basis of multiple autapomorphies. The fourth specimen has several cranial features as in typical terrestrial extant hynobiids, stoutly ossified bony skeleton and moderately well-developed dorsal tail fin, indicating that this species is semiaquatic in life, an ecological preference that characterizes some extant hynobiids but has not yet been identified in Mesozoic salamanders. Inspired by phenotypic variations between the fourth and the holotype specimens of “*L*. *daohugouensis*”, in urodeles and early amphibians, we recognize that both the number and variability of caudosacral vertebrae are evolutionarily reduced likely constrained by different fertilization modes. We also conducted morphology-based cladistic analyses for all hynobiids at the generic level. Our results show that the new taxon is a stem hynobiid and predate the estimated time for the origin of total-group hynobiids at least 8 Myr earlier than the hypothesized ∼157 Ma from molecular studies (Chen et al., 2015) and allow us to establish a clade for both stem and crown hynobiids to trace the morphological evolutionary history of this early-branching salamander clade.

## Results

### Systematic paleontology

Class: Amphibia Linnaeus, 1758

Order: Urodela Duméril, 1806

Suborder: Cryptobranchoidea Dunn, 1922

PANHYNOBIA, *nomen cladinovum*

**Registration number**—415.

**Definition**—The largest total clade containing *Neimengtriton daohugouensis*, comb. nov. (Middle Jurassic); *Linglongtriton daxishanensis* (Late Jurassic); and *Liaoxitriton zhongjiani*, *Nuominerpeton aquilonaris*, and *Regalerpeton weichangensis* (all Early Cretaceous) and *Hynobius* (extant), but not *Andrias* (extant) and *Cryptobranchus* (extant). This is a maximum-total-clade phylogenetic definition. Abbreviated as max total ∇ (*Liaoxitriton* & *Linglongtriton* & *Neimengtriton* & *Nuominerpeton* & *Regalerpeton* & *Hynobius* ∼ *Andrias* & *Cryptobranchus*) (see de Queiroz et al., 2020).

**Etymology**—*Pan*, Gr. “all, total” indicating reference to a total clade + *hynobia*, means Hynobiidae.

**Reference phylogeny**—The reference phylogeny is Figure 6 in this article.

**Composition**—*Batrachuperus*, *Hynobius*, *Liaoxitriton*, *Linglongtriton*, *Liua*, *Neimengtriton*, *Nuominerpeton*, *Onychodactylus*, *Pachyhynobius*, *Paradactylodon*, *Parahynobius*, *Protohynobius*, *Pseudohynobius*, *Ranodon*, *Regalerpeton*, and *Salamandrella*.

**Diagnosis**—The clade is characterized by possession of the following four apomorphies (numbers in parentheses denote character numbers and polarities in supplemental information) as inherited by *Neimengtriton* and *Hynobius*: optic foramen opens at the posterior border of orbitosphenoid (54-0); dorsal and ventral crests of humerus well developed (88-1); femoral trochanter forming a twig-like projection from the shaft (89-1); and mesopodium ossified (108-1).

**Comments**—Our hypothesized content for clade Panhynobia is based on the findings of cladistic analyses in this study, which show that the above-listed Chinese Mesozoic taxa are placed along the stem leading to a monophyletic crown Hynobiidae. *Pangerpeton* was recovered as “the basal-most stem hynobiid” (Jia and Gao, 2019: p. 13), whereas based on increased sampling of both characters and taxa in Cryptobranchoidea, *Pangerpeton* was instead recovered here as a basal taxon of the total group Cryptobranchoidea. Jia and Gao (2019) analysis was consistent with some earlier studies (Zhang et al., 2009; Gao and Shubin, 2012; Jia and Gao, 2016b; Rong, 2018) in finding *Iridotriton* is related to hynobiids or some combination of stem hynobiids, yet because *Iridotriton* is so poorly known it remains frustratingly unclear whether the genus lies within or outside of clade Panhynobia. For that reason, we conservatively exclude *Iridotriton* from Panhynobia. For the same reason as *Iridotriton*, we also exclude *Kiyatriton* from Siberian Russia (Skutschas, 2014, 2016). Based on the published original description (Zhang and Fan, 2001), we regard the Chinese Middle Jurassic “*Voldotriton* [sic] *sinensis*” as potentially hynobiid-like, but because many features of the holotype and only known skeleton were misinterpreted and the specimen has been lost (Gao et al., 2013), we exclude that problematic species from clade Panhynobia. Finally, although the Early Cretaceous *Laccotriton subsolanus* and *Sinerpeton fengshanensis* from the Jehol Biota in northern China have been regarded as hynobiid-like salamanders (e.g., Gao and Shubin, 2001; Wang and Evans, 2006a; Jia and Gao, 2016a) but that has not been tested cladistically; for that reason, we also refrain from including those species in clade Panhynobia.

Genus *Neimengtriton*, gen. nov.

**Type and only known species**—*Neimengtriton daohugouensis*, comb. nov.

**Diagnosis**—As for the type and only known species.

**Etymology**—“Neimeng” (Pinyin), referring to the Nei Mongol (Inner Mongolia) Autonomous Region where the Daohugou locality is located; “triton” (Gr.), suffix commonly used for salamander generic names.

Species *Neimengtriton daohugouensis*, comb. nov.

**Holotype**—IVPP V13393, natural mold of articulated skeleton with stain of body outline, exposed in ventral view on a single slab (type of “*Liaoxitriton daohugouensis*” by original designation).

**Figure 1:**
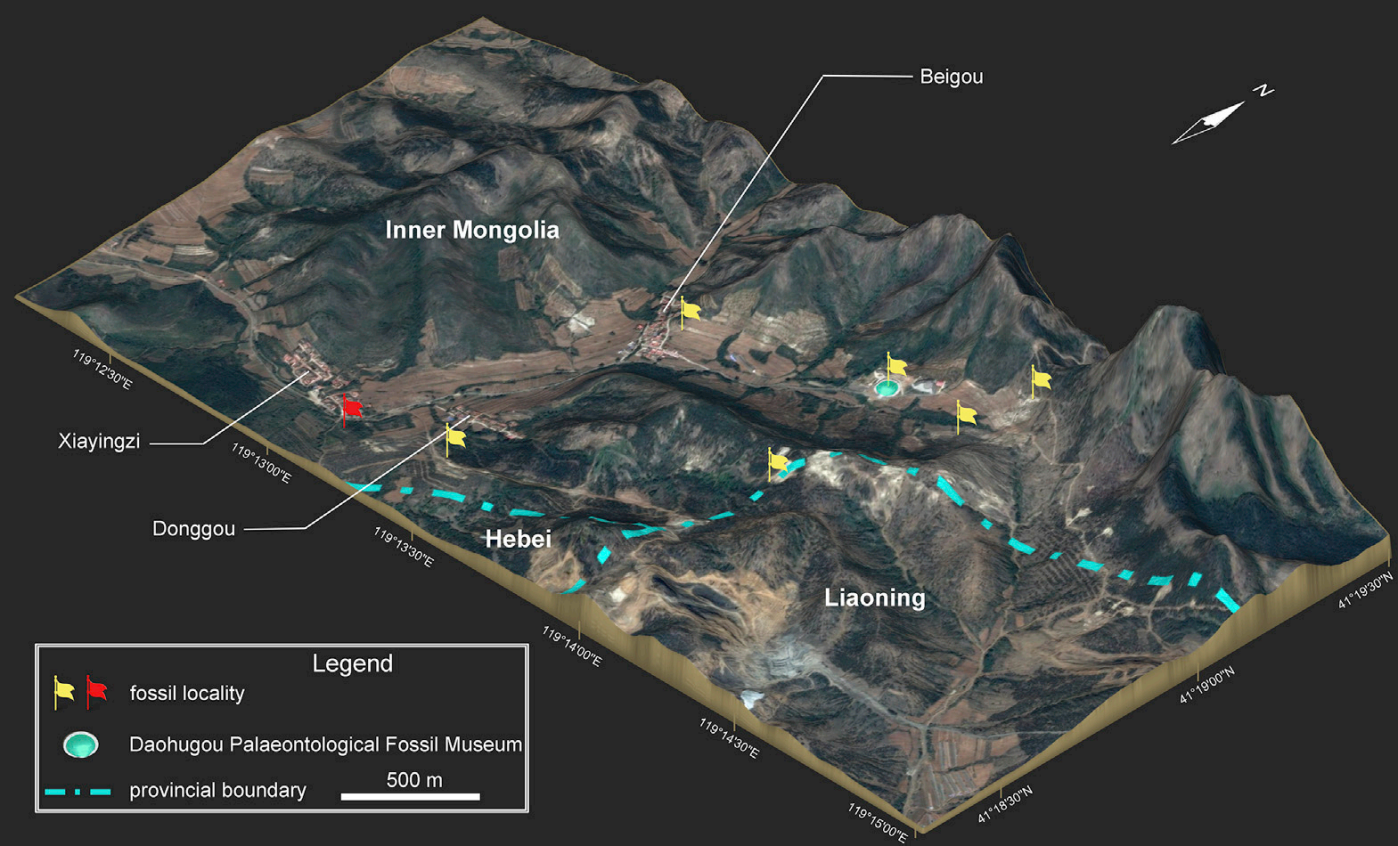
Three-dimensional satellite map showing sublocalities of the Middle Jurassic Daohugou locality around the settlements of Xiayingzi, Donggou, and Beigou (collectively called Daohugou Village), Ningcheng County, Inner Mongolia, China Red flag marks the sublocality from where PKUP V0515 was collected. Yellow flags mark other fossil sublocalities around the Daohugou area (localitiy information see Dong and Huang, 2011; Huang et al., 2015).

**Type locality and horizon**—Daohugou locality, Ningcheng County, Inner Mongolia, China; Middle Jurassic (Bathonian) Haifanggou Formation (Figure 1; see STAR Methods).

**Paratypes**—IVPP V14062, natural mold of skeleton exposed in ventral aspect (Wang, 2004a) but neither described nor figured.

**Figure 2:**
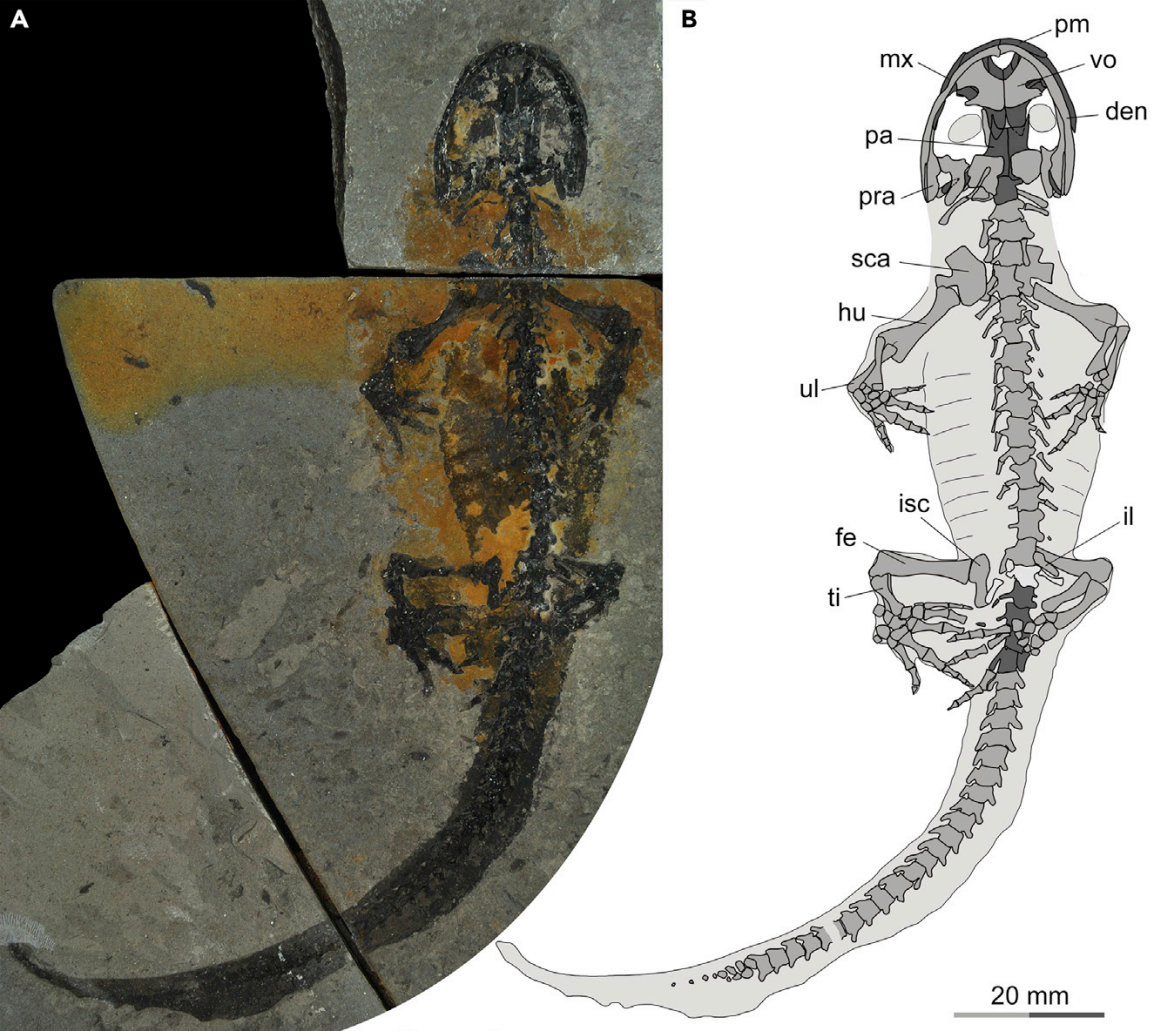
Photograph and line drawing of the newly referred specimen (PKUP V0515) of the stem hynobiid *Neimengtriton daohugouensis* comb. nov., as exposed in slab A (A) Photograph. (B) Line drawing. In the latter image, skull roof bones and caudosacral vertebrae are shaded dark gray, sacral vertebra is shaded whitish, rest of skeleton is shaded medium gray, and soft tissue traces are shaded light gray. See supplemental information for abbreviations.

**Figure 3:**
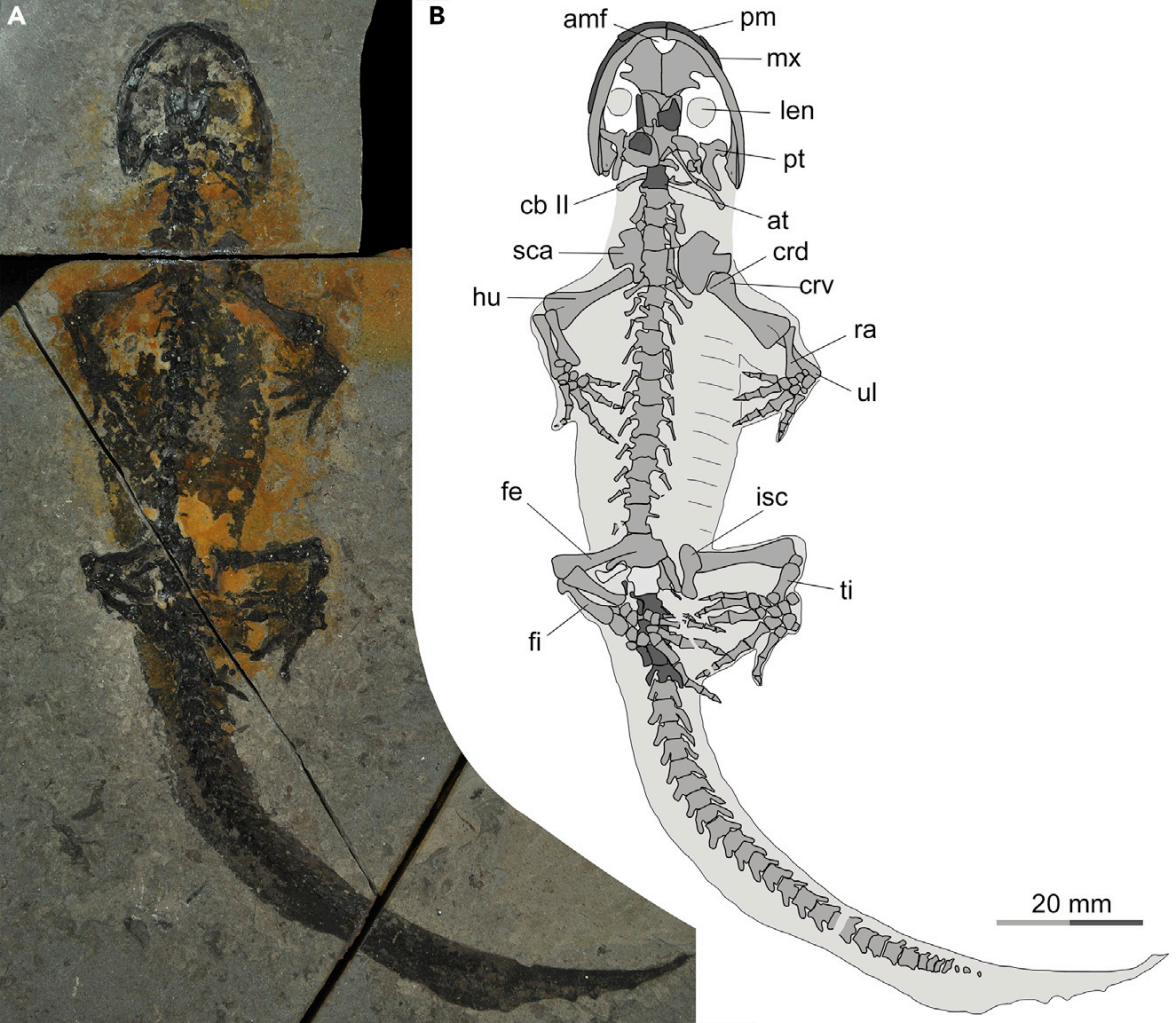
Photograph and line drawing of the newly referred specimen (PKUP V0515) of the stem hynobiid *Neimengtriton daohugouensis* comb. nov., as exposed in slab B (A) Photograph. (B) Line drawing. In the latter image, skull roof bones and caudosacral vertebrae are shaded dark gray, sacral vertebra is shaded whitish, rest of skeleton is shaded medium gray, and soft tissue traces are shaded light gray. See supplemental information for abbreviations.

**Figure 4:**
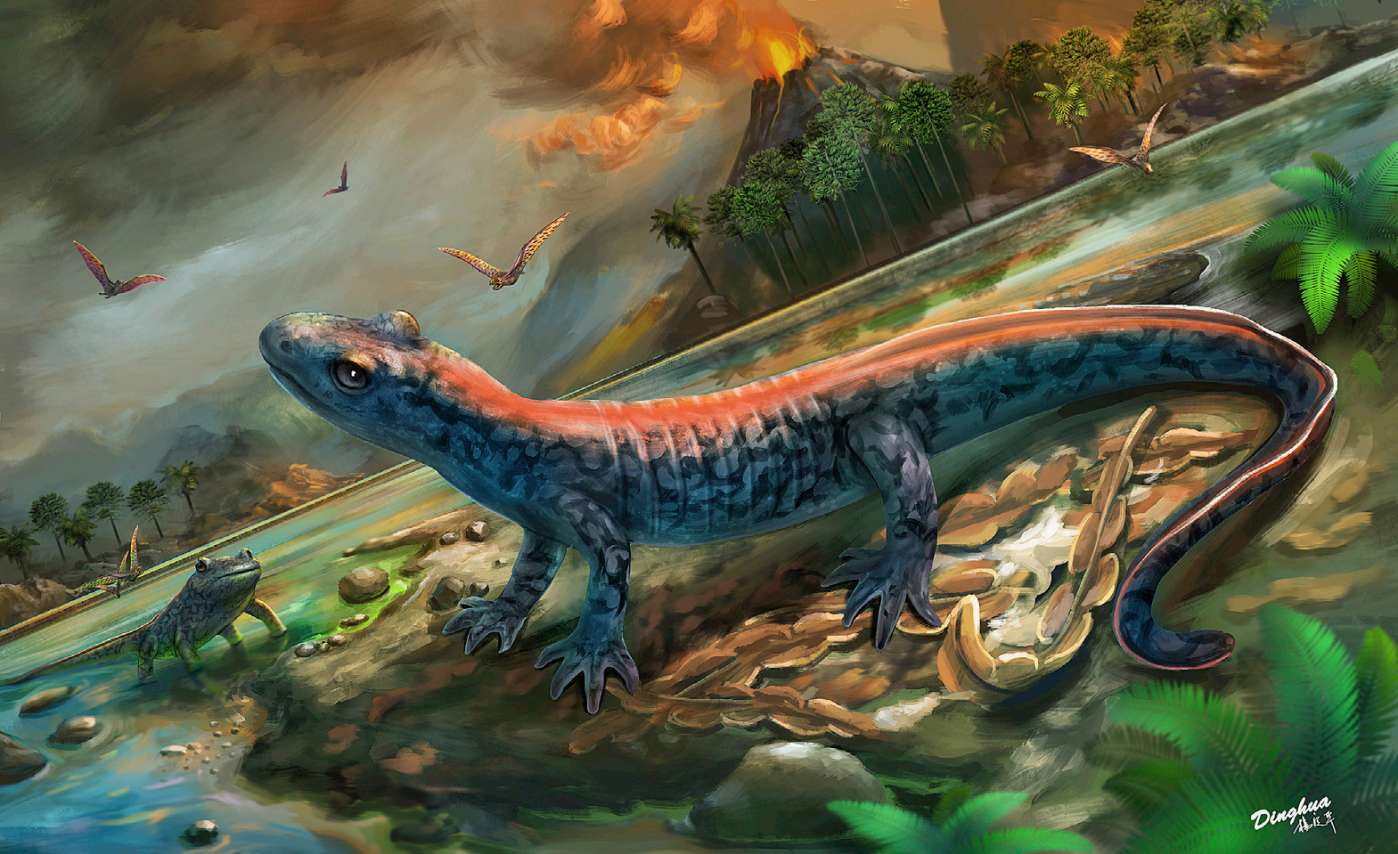
Restoration of *Neimengtriton daohugouensis* comb. nov. Showing the stem hynobiid is semiaquatic living nearby the water bodies during the Middle Jurassic Bathonian at the Daohugou locality, Inner Mongolia, China.

**Referred specimens**—IVPP V14057, reported but not described or figured by Sullivan et al. (2014); outline drawing of its scapulocoracoid was provided by Zhang et al. (2009; figure 4C). Newly referred specimen PKUP V0515, articulated and nearly complete bony skeleton, preserved on part and counterpart slabs, with impressions of soft tissues including body outline, tail fin, costal grooves, and eye lenses (Figures 2, 3, and 4; supplemental information; Video S1). Considering that PKUP V0515 preserves bone, it clearly came from a horizon differs from that of both IVPP V13393 and V14062, both of which are natural molds.

Video S1. Micro-CT rendered skull and craniocervical region of *Neimengtriton daohugouensis* comb. nov. based on PKUP V0515, related to figures 2 and 3

**Emended diagnosis**—Middle Jurassic stem hynobiid differing from all other known panhynobians in having the following autapomorphies: parietal elongate; palatal process of pterygoid expanded bilaterally; vomer extended posteriorly, reaching close to the middle transverse plane of the cranium; cultriform process of parasphenoid anteroposteriorly shortened and anteriorly broadened; and scapular blade shortened to slightly more than one-half width of coracoid. Differs further from other panhynobians in the following unique combination of features: metamorphosed; snout broadly rounded in dorsal or ventral view; lacrimal enters into orbit, but not into external naris; frontoparietal fontanelle open; vomerine tooth row transversely arranged and located close to the posterior edge of vomer; prootic fused with opisthotic-exoccipital complex as a single unit; footplate of stapes enlarged; stapedial foramen present; caudosacrals four or seven in number; two centralia ossified in both manus and pes; postminimus ossified in pes; metacarpals II and III not expanded anteroposteriorly; phalangeal formulas are 2-2-3(4)-2, or 2-2-4-3 for the manus and 2-2(3)-3-4-2 for the pes.

Description of PKUP V0515 was moved to supplemental information.

## Discussion

### Taxonomy and palaeobiology of *N.daohugouensis* comb. nov

The nominal taxon “*L. daohugouensis*” was established and classified into *Liaoxitriton* sensu stricto (Wang, 2004a) 17 years ago when there were fewer known stem hynobiid fossil specimens and consequently poor appreciation of osteological patterns in both crown and stem hynobiids. Not surprisingly, none of the features that initially were considered diagnostic for *Liaoxitriton* sensu lato (e.g., skull bones lacking sculpture, vomers meet at midline and with an anteromedial fenestra anteriorly, 15 or 16 presacrals; Dong and Wang, 1998; Wang, 2004a; 2004b; Wang et al., 2008) or features claimed as synapomorphies uniting “*L. daohugouensis*” and *L*. *zhongjiani* (e.g., short and approximately transverse vomerine tooth rows; Sullivan et al., 2014) are broadly distributed among other fossil (e.g., *Nuominerpeton* in Jia and Gao, 2016a) and extant (e.g., *Batrachuperus* in Jia et al., 2019) hynobiids or are developmental artifacts (e.g., mesopodials partially ossified). Our morphological study of PKUP V0515 by micro-computed tomography scan (see supplemental information) provides details on the dermal skull roof, suspensorium, braincase, mandible, autopodium of both the manus and pes, and tail fin that were unclear or not available in the holotype (IVPP V13393). We recognized several autapomorphies of *N*. *daohugouensis* (see Emended diagnosis; Figures S1 and S2) and two other diagnostic features that are plesiomorphic for Urodela and never have been reported in other stem hynobiids, namely the presence of a stapedial foramen and the presence of an ossified postminimus in the pes (Figures S3 and S4). We agree with certain morphological differences between *N*. *daohugouensis* and *L*. *zhongjiani* as recognized in previous studies (Wang, 2004a; 2004b; Wang and Evans, 2006a: table 2; Wang et al., 2008; Sullivan et al., 2014), including rostrum wide and rounded in *N*. *daohugouensis* vs. narrower and subpointed in *L*. *zhongjiani*; vomerine tooth rows oriented anterolaterally in *N*. *daohugouensis* vs. posterolaterally in *L*. *zhongjiani*; and metacarpal II not anteroposteriorly expanded in *N*. *daohugouensis* vs. expanded in *L*. *zhongjiani*. But, other previously proposed differences between these two taxa are rejected because they are potentially dependent on ontogenetic age (relative height of coronoid flange on prearticular, relative robustness of ribs, counts for postsacral ribs)—an important consideration given that fully grown individuals of both *L*. *zhongjiani* and *N*. *daohugouensis* have yet to be described (Dong and Wang, 1998; Wang, 2004a; 2004b; Wang and Evans, 2006a; Wang et al., 2008; this study). Along with the nonsister group relationship (see below) recovered for *N*. *daohugouensis* and *L*. *zhongjiani*, we strongly support transferring “*L*. *daohugouensis*” from *Liaoxitriton* sensu stricto into the genus *Neimengtriton*. As a result, our study shows that there are no longer any vertebrate genera in common between the Yanliao and Jehol biotas.

As mentioned previously, hynobiids living today are predominantly metamorphic except for some populations of *Batrachuperus londongensis* that are the only facultative neotenes (Fei and Ye, 2016). Neotenic *B*. *londongensis* are aquatic at the adult stage, whereas postmetamorphosed extant hynobiids are diverse in ecological preferences, with adults outside of the breeding season living in water (e.g., *Batrachuperus*, *Pachyhynobius*), on land (e.g., *Hynobius*, *Protohynobius*), or alternating between aquatic and terrestrial habitats (*Onychodactylus* and *Ranodon*; Kuzmin and Thiesmeier, 2001; Poyarkov et al., 2012; Fei and Ye, 2016). In other words, ecological preferences are not correlated with life history strategies in extant hynobiids. Among Mesozoic panhynobians and hynobiid-like taxa, *Regalerpeton* is the only known neotene, recognized as such by its possession of several larval features in adult specimens, including gill rakers, external gills, and an anteromedial directed palatal process of the pterygoid (Rong, 2018). Based largely on the absence of these larval features and presence of an anterolaterally directing palatal process of the pterygoid in adult specimens, several other taxa (*Laccotriton*, *Liaoxitriton*, *Linglongtriton*, *Nuominerpeton*, and *Neimengtriton*) are considered metamorphic (e.g., Dong and Wang, 1998; Gao et al., 1998; Gao and Shubin, 2001; Wang 2004a; Jia and Gao, 2016a, 2019). The Early Cretaceous *Sinerpeton* was originally proposed as neotenic (Gao and Shubin, 2001) by the presence of ossified ceratobranchials in adult specimens. However, it is now clear that ossified ceratobranchials are present among hynobiids in both neotenic (e.g., *Batrachuperus londongensis*) and metamorphic (e.g., *Nuominerpeton aquilonaris*) taxa and should not be used as a reliable indicator to neoteny. Instead, the presence of an anterolaterally directing palatal process of the pterygoid and the absence of aforementioned larval features suggest that *Sinerpeton* is metamorphic. The Late Jurassic *Pangerpeton* originally was described as metamorphic based on the absence of external gills (Wang and Evans, 2006b) but was later suggested to be neotenic without any supporting evidence provided (Wang et al., 2016).

Paleoecological interpretations for these metamorphic fossil taxa are rare, except that *Nuominerpeton* was interpreted as terrestrial, based on its well-developed limbs, extensive ossified mesopodials, and shallow tail fin (Jia and Gao, 2016a). Fortuitously, PKUP V0515 has an intact tail exposed in lateral view and shows that the dorsal caudal fin arises anteriorly from the base of the tail, and the ventral fin arises behind the cloaca (Figures 2 and 3). The upper edge along the posterior portion of the dorsal fin is indented by shallow wrinkles, indicating that portion of the fin was thin and flexible. By contrast, surfaces along the lower part of the dorsal fin are smooth, suggesting that portion was fleshy and not pliable, presumably because it was supported by bundles of myotomic muscle as in extant salamanders (Duellman and Trueb, 1986). The tall and flexible dorsal tail fin of *Neimengtriton* seems ideal for swimming and steering when in water, but is sufficiently thin and low not to be a hindrance when walking on land. Interestingly, this taxon has several features that are widely present in extant terrestrial hynobiids (Jia et al., 2021), including skull relatively short anteroposteriorly with a rounded snout, many vomerine teeth with the vomerine teeth rows spanning most the transverse dimension of the palate, and limbs extensively ossified (supplemental information). Based on the morphology of the tail fin and the aforementioned features in the cranial and appendicular skeleton, we interpret *Neimengtriton* as a semiaquatic salamander (Figure 4), broadly analogous to semiaquatic extant hynobiids such as *Onychodactylus* (Poyarkov et al., 2012) or *Ranodon* (Fei and Ye, 2016). To date, the vast majority of salamander specimens discovered from the lacustrine deposits at the Daohugou locality belong to two neotenic species: the cryptobranchoids *Chunerpeton tianyiensis* and *Jeholotriton paradoxus* (e.g., Wang, 2000; Gao and Shubin, 2003). Our identification of the contemporaneous metamorphic *N. daohugouensis* as semiaquatic reveals a more complete paleoecomorphic space for early cryptobranchoids awaits to be quantatively investigated.

### Reduction of caudosacral vertabra and its correlation with fertilization modes

In extant salamanders, both intraspecific and interspecific phenotypic variations within a clade have been shown to be correlated with external (e.g., ecological parameters; Wake and Dresner, 1967; Blaustein and Johnson, 2003; Wang et al., 2016) and/or internal constraints (e.g., life history strategies; Hanken, 1984; Shubin et al., 1995; Wake, 2009; Arntzen et al., 2015; Bonett and Blair, 2017; Ledbetter and Bonett, 2019) on phenotypic diversity. However, rare attention was paid to phenotypic variations in Mesozoic salamanders. When comparing with the subadult holotype specimen of *N. daohugouensis*, the almost fully grown specimen PKUP V0515 not only shows phenotypic variations that are related to postmetamorphic development (increased ossification in basibranchial II and the carpals and tarsals; see STAR Methods), but also two variations of phenotypes that are formed early during embryonic development: phalangeal formulas and the counts of caudosacral vertebrae. For phalangeal counts in *Neimengtriton*, both the manus and pes in PKUP V0515 have phalangeal formulas that differ from those in the holotype (Figures 2, 3, and S4): 2-2-4-2 in the left manus and 2-2-4-3 in the right manus in PKUP V0515 vs. 2-2-3-2 in the preserved left manus in the holotype and 2-3-3-4-2 in both the left and right pes in PKUP V0515 vs. 2-2-3-4-2 in the preserved left pes in the holotype. The phalangeal formulas in the manus and pes in PKUP V0515 of *N*. *daohugouensis* have never been documented from the salamander fossil record, and its implications are being addressed in another ongoing project regarding the mechanism of phenotypic variations on limb structures of salamanders to be published elsewhere. For caudosacral counts in *Neimengtriton*, the holotype was interpreted as seven (Jia and Gao, 2019), and there are only four in PKUP V0515. As compared to appendicular skeleton, much less attention has been devoted to deciphering the underlying mechanisms behind variation in the count of caudosacrals (e.g., Litvinchuk and Borkin, 2003; Arntzen et al., 2015), because 1) for fossil salamander taxa represented by skeletons, typically only a few specimens preserving the caudosacral region have been adequately described and figured (e.g., for *Neimengtriton* only two, the holotype and PKUP V0515, of the four known specimens have been described and figured); 2) descriptions for most previously established fossil taxa (e.g., *Beiyanerpeton*, *Chunerpeton*, *Jeholotriton*, *Laccotriton*, *Liaoxitriton*, *Pangerpeton*, *Regalerpeton*, and *Sinerpeton*) did not or wrongly differentiate caudosacrals from caudal vertebrae (see STAR Methods); 3) identification of caudosacral vertebrae as a morphologically and functionally distinct region did not start until the 1960s by Wake (1963). Therefore, even Francis (1934), in his classic monograph on the anatomy of extant *Salamandra,* did not explicitly recognize caudosacrals as distinct from the caudals.

**Figure 5:**
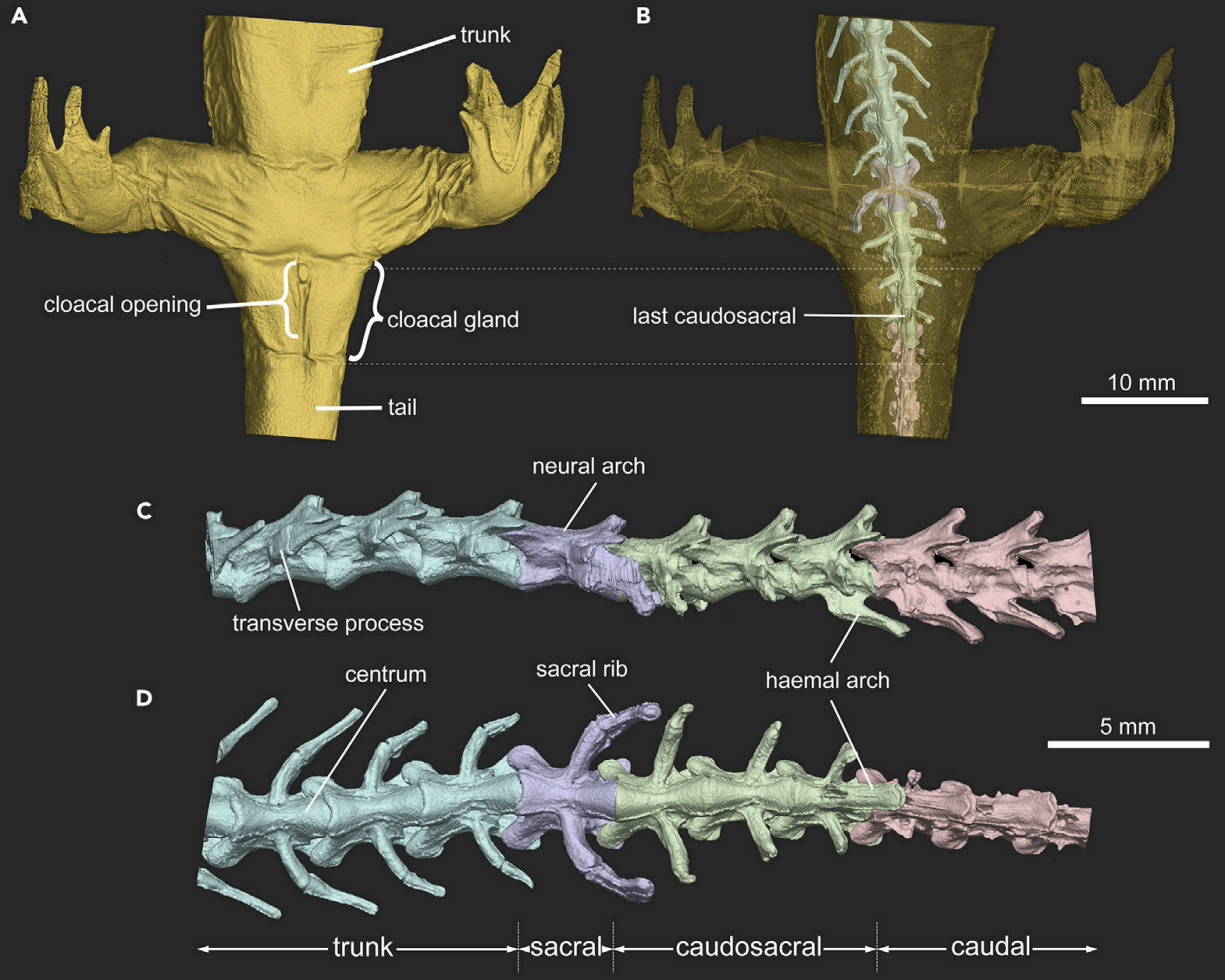
Micro-CT rendered reconstructions of tail base region in an alcohol-preserved, adult specimen (ZMNH AA871) of the extant hynobiid *Hynobius amjiensis*, showing morphology and positional correlation between cloaca and vertebrae (A) External surface of tail base region in ventral view, showing cloaca and trunk-tail junction. (B) The same view, but with soft tissue digitally rendered translucent and with pelvic girdle and hind limbs digitally removed to show the vertebrae. (C) Color-coded posterior trunk-sacral-caudosacral-anterior tail vertebrae in left lateral view. (D) Color-coded posterior trunk-sacral-caudosacral-anterior tail vertebrae in ventral view. Following Wake (1963), the last caudosacral is identified as the first postsacral vertebra bearing a complete haemal arch; this individual has three caudosacrals.

Our comparisons suggest a reduction in caudosacral vertebral counts through time in the evolution of salamanders and probably amphibians in general. The Carboniferous to Early Jurassic temnospondyls have six to nine caudosacrals (Table 1). The Middle to Late Jurassic salamanders, with the exception of *Jeholotriton* (two caudosacrals), tend to have more caudosacrals (three to seven) than Cretaceous salamanders (three or four), which, in turn, have more caudosacrals than extant salamanders (one to three; Table 1). This raises the questions of what drives the reduction of caudosacral numbers in salamanders and what impact or benefit might that reduction confer to salamanders? We posit that reduction and variability in caudosacral counts are linked to fertilization modes. The caudosacrals lie directly above the cloacal region in modern salamanders (e.g., Wake and Dresner, 1967; Jia et al., 2019; Figure 5), are functionally different from caudal vertebrae (see below), and are considered part of the trunk region (Duellman and Trueb, 1986). Both the cloaca and its associated structures are firmly attached by large amounts of connective tissue to the ventral side of the centrum of the last caudosacral and its haemal arch (Wake and Dresner, 1967). In addition, the M. caudali-femoralis and M. ischio-caudalis attach to the intermyotomal septum that is a continuation of the haemal arch of the last caudosacral vertebra, and the M. caudali-pubo-ischio-tibialis originates on the margin of that arch (Francis, 1934; Wake and Dresner, 1967). Therefore, the location of the last caudosacral vertebra can be a reliable indicator of the position of the cloaca in both extant and fossil specimens. As mentioned earlier, extant salamanders have two distinct modes of fertilization: the plesiomorphic external fertilization in the suborders Cryptobranchoidea and Sirenoidea and the derived internal fertilization mode in the suborder Salamandroidea (e.g., Duellman and Trueb, 1986). Internal fertilization is widely considered to have evolved only once among salamanders as a synapomorphy for the suborder Salamandroidea (e.g., Sever, 1991). Recent studies have argued that the divergence between Cryptobranchoidea and Salamandroidea occurred no later than the Oxfordian in the early Late Jurassic (e.g., Gao and Shubin, 2012; Jia and Gao, 2016b) and, likely, even earlier in the Middle Jurassic as suggested in some molecular dating analyses (e.g., Zhang and Wake, 2009), which implies internal fertilization arose around the same time.

**Table 1:** Variations in caudosacral vertebral counts in Mesozoic salamanders and temnospondyls known from informative skeletons

Age	Taxon name	Caudosacral #	Reference
**Cryptobranchoidea**			
Cretaceous	*Sinerpeton fengshanensis*	–	Gao and Shubin (2001)
Cretaceous	*Laccotriton subsolanus*	–	Gao and Shubin (2001)
Cretaceous	*Liaoxitriton zhongjiani*	–	Dong and Wang (1998)
Cretaceous	*Nuominerpeton aquilonaris*	3 or 4	Jia and Gao (2016a)
Cretaceous	*Regalerpeton weichangensis*	4	Rong (2018)
Jurassic	*Linglongtriton daxishanensis*	5	Jia and Gao (2019)
Jurassic	*Neimengtriton daohugouensis*	4 or 7	Jia and Gao (2019); this study
Jurassic	*Pangerpeton sinensis*	4	Wang and Evans (2006b); this study
Jurassic	*Jeholotriton paradoxus*	2	Wang and Rose (2005)
Jurassic	*Chunerpeton tianyiensis*	4	Gao and Shubin (2003); Wang et al. (2016)
**Salamandroidea**			
Cretaceous	*Valdotriton gracilis*	3	Evans and Milner (1996)
Jurassic	*Beiyanerpeton jianpingensis*	3	Gao and Shubin (2012)
Jurassic	*Qinglongtriton gangouensis*	4	Jia and Gao (2016b)
**Karauridae**			
Jurassic	*Karaurus sharovi*	5	Ivachnenko (1978)
**Temnospondyli**			
Carbonif.	*Dendrerpeton acadianum*	+6	Holmes et al. (1998)
Permian	*Doleserpeton annectens*	7	Sigurdsen and Bolt (2010)
Permian	*Eryops megacephalus*	9	Pawley (2006)
Carbonif.	*Eoscopus lockardi*	9	Pawley (2006)
Triassic	*Mastodonsaurus giganteus*	9	Pawley (2006)
Jurassic	*Siderops kehli*	9	Warren and Hutchinson (1983)
Permian	*Archegosaurus decheni*	6	Witzmann and Schoch (2006)

The number of caudosacral vertebrae identified by us following the criteria of Wake (1963).

**Figure 6:**
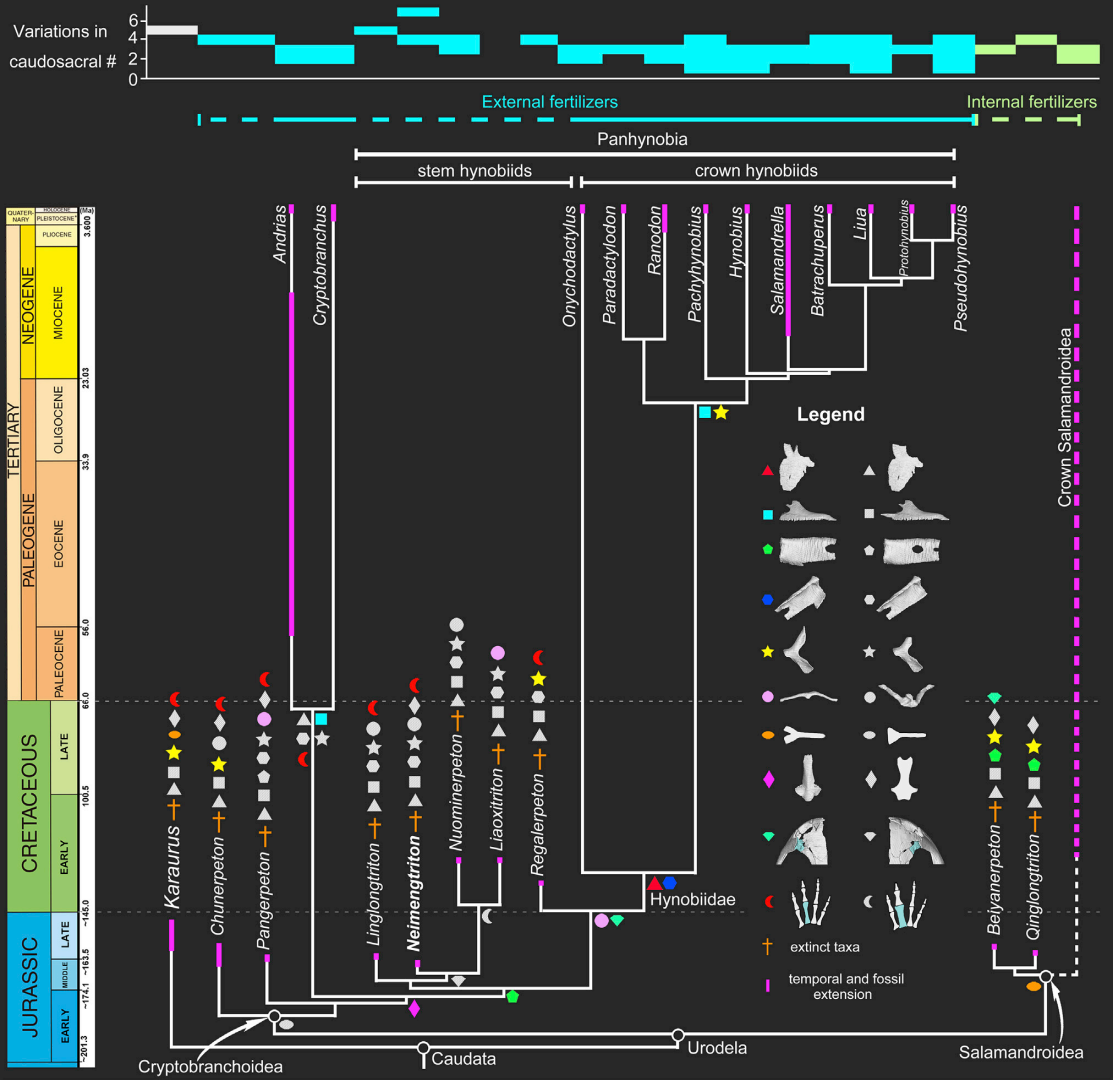
Time-calibrated cladogram showing phylogenetic relationships of the clade Panhynobia (stem + crown hynobiids) and related salamanders, along with diagrammatic summaries of variations in caudosacral vertebrae counts Fossil taxa of Cryptobranchoidea and Salamandroidea are tentatively regarded (dotted horizontal lines) as external and internal fertilizers, respectively, based on fertilization modes with their living relatives (solid horizontal lines). Note the sister-group relationship between the clade of *Beiyanerpeton* and *Qinglongtriton* and the crown salamandroids (dashed line) are derived from Figure S5 in supplemental information. Distributions of ten osteological characters important for resolving relationships among panhynobians are taken from Jia and Gao (2019) and this study, and are mapped onto the tree using color-coded symbols, as follows: triangle (anterior edge of nasal in dorsal view; red = bifurcated vs. white = not bifurcated); square (anterior process of maxilla in lateral view: light blue = anteroposteriorly short vs. white = elongate and slender); pentagon (orbitosphenoid in lateral view, two characters: green = optic foramen opens in posterior border of orbitosphenoid vs. white = foramen opens within orbitosphenoid; green = oculomotor foramen opens behind orbitosphenoid vs. white = foramen opens at posterior border of orbitosphenoid); hexagon (orbitosphenoid in dorsomedial view: dark blue = anteroventral process of orbitosphenoid present vs. white = absent); star (pterygoid in ventral view: yellow = palatal process equal to or longer than quadrate process vs. white = palatal process shorter than quadrate process); circle (basibranchial II in dorsal view: light pink = non-anchor shaped vs. white = anchor shaped); ellipse (rib head: orange = bifurcate vs. white = unicapitate); rhombus (parasphenoid in ventral view: dark pink = cultriform process anteriorly constricted bilaterally vs. white = cultriform process anteriorly expanded bilaterally); diamond (lacrimal region of the skull roof in dorsal view; green = lacrimal enters into both the naris and orbit vs. white = lacrimal enters into orbit only); crecent (digits in dorsal view: red = metacarpal II not expanded vs. white = metacarpal II expanded anteroposteriorly).

When mapped onto a phylogenetic tree (Figure 6), caudosacral counts have a larger range of variation in basal cryptobranchoids (two to seven collectively for *Chunerpeton*, *Jeholotriton* [not depicted], *Linglongtriton*, *Neimengtriton*, and *Pangerpeton*) compared with basal salamandroids (three or four collectively for *Beiyanerpeton* and *Qinglongtriton*). Although not depicted in Figure 6, the Early Cretaceous *Valdotriton* (three caudosacrals) also falls within the basal salamandroid range. A major implication of those differences is that the position of the cloaca is more flexible along the postsacral region in basal cryptobranchoids than in basal salamandroids. The holotype and PKUP V0515 of the stem hynobiid *Neimengtriton daohugouensis* are notable for showing that the position of the cloaca can vary intraspecifically over a distance of at least three caudosacrals.

Our data suggest that caudosacral counts and, by implication, cloacal positions have changed at least twice during the evolution of crown salamanders: initially with the origin of internal fertilization in salamandroids and again during subsequent evolution within salamandroids and cryptobranchoids. In the external-fertilizing cryptobranchid *Andrias davidianus*, during oviposition, the female deposits eggs in her den after the male butts her sides with his head and then, with their tails crossed, the male fertilizes the eggs by ejaculating his sperm into the water (Luo et al., 2018). By contrast, in internal fertilizing species, the male either deposits spermatophores (gelatinous structure with a cap of sperm and a base consists of viscous secretions from the male cloaca) directly into the female's cloaca or places the spermatophore on the substrate near the female, who then positions herself over the spermatophore and lowers her cloaca so that most of the spermatophore or only the sperm cap of the spermatophore is picked up by her cloacal lips. Regardless of how the female internal-fertilizing salamander takes receipt of the spermatophore, it is stored in her spermatheca (tubules imbedded in loose connective tissue in the roof of the cloaca at the opening of the oviduct) within her cloaca (Duellman and Trueb, 1986). For externally fertilizing species, cloacal position would seem to have a trivial effect on the efficiency of successful fertilization, as long as the male and female deposit their spermatophore and eggs, respectively, in close enough proximity to fertilize the eggs. Because there is no constraint on the position of the cloaca, caudosacral counts can vary without negatively impacting fertilization. For internally fertilizing species, it has been argued that their suite of apomorphic cloacal glands in males (such as dorsal pelvic glands, male dorsal or vent glands, and Kingsbury's glands) that assist in producing spermatophores, the apomorphic spermatheca in females for internal storage of sperm, and complex courtship behaviors (e.g., tail fanning, wagging, tapping, undulating, tail straddling/nudging walk), evolved in tandem and simultaneously with the origin of Salamandroidea (Sever, 1991; Houck and Arnold, 2003). Because of the need for a precise match between the male and female cloaca and for fine control of muscles at the tail base for tail display, internal fertilization in salamanders imposes constraints on the anatomy and position of the cloaca, which, in turn, presumably restricts variability in caudosacral counts to a narrower span along the base of the tail.

### Sequential evolution of morphological characters of the Hynobiidae

Given that the widely accepted basal position of the Hynobiidae, the phylogenetic importance of the family cannot be over emphasized in understanding the historical evolution of urodeles. Our cladistic analyses based on a data matrix designed for Cryptobranchoidea with updates from PKUP V0515 and our accumulating knowledge on the morphology of both fossil and extant hynobiids enable us to understand the evolutionary history of all hynobiids at the generic level. Our results (Figures 6 and S5; supplemental information) reinforce the arguments of Jia and Gao (2019) that five fossil taxa (*Liaoxitriton*, *Linglongtriton*, *Neimengtriton*, *Nuominerpeton*, and *Regalerpeton*) are stem hynobiids, with the first four taxa forming a sister clade to (*Regalerpeton* + crown hynobiids); however, both *Chunerpeton* and the hynobiid-like *Pangerpeton* are recovered as successive outgroups of crown Cryptobranchoidea; the two basal salamandroids *Beiyanerpeton* and *Qinglongtriton* are closely related to the modern salamandroid *Ambystoma* (Figure S5) and collectively form as the sister clade to total Cryptobranchoidea. Excluding *Chunerpeton* from Cryptobranchidae as proposed in the original study (Gao and Shubin, 2003) may collapse the calibration point for crown group Cryptobranchidae, but our recognition of *Neimengtriton* as a stem hynobiid reinforce the previous hypotheses that the divergence of the Hynobiidae-Cryptobranchidae clades had taken place no later than the Middle Jurassic Bathonian time (168.3–166.1 Mya; Jia and Gao, 2019). In contrast to the estimated Late Jurassic (157.1 Mya in Chen et al., 2015) origin of total-group hynobiids inferred from molecular evidence, our study pushes back in time the calibration point for the origin of panhynobians for at least 8.6 Myr.

Our study shows that all stem and crown-group hynobiid species share four salient synapomorphies: optic foramen open at the posterior border of orbitosphenoid (54-0); dorsal and ventral crests of humerus well developed (88-1); femoral trochanter forming a twig-like projection from the shaft (89-1); and mesopodium ossified (108-1). Among stem hynobiids, *Liaoxitriton* was not recovered as the sister group taxon to *Neimengtriton* but instead is united with *Nuominerpeton* by three synapomorphies: vomerine teeth located in the middle part of vomer (37-1); the inner and outer branch of vomerine teeth row similar in length (39-1); and metacarpal II expanded (95-0). *Neimengtriton* and *Linglongtriton* are two successive outgroups of (*Liaoxitriton* + *Nuominerpeton*), and each of them is united with (*Liaoxitriton* + *Nuominerpeton*) by two synapomorphies: maxilla/nasal contact present (7-1) and lacrimal exters the orbit (12-1) for the former, and posterolateral border of vomer deeply notched for choana (36-2) and dentary groove present (61-0) for the latter. On the other hand, the lacrimal enters both the naris and the orbit (12-3) in *Regalerpeton* and the crown hynobiids. The crown Hynobiidae was argued to have no synapomorphies, but here, we recognized six synapomorphies, including nasal anterior border notched to receive the alary process of premaxilla (15-1); quadrate distally expanded (28-1); anteroventral process of orbitosphenoid present (53-0); prootic fused with opisthotic and exoccipital (56-2); caudosacral vertebrae count reduced to less than or equal to three (82-2); and height of ossified scapular shorter than the proximodistal length of coracoid portion of scapulocoracoid (86-0). Crown ward to *Onychodactylus*, the clade containing *Ranodon* and *Hynobius* is supported by two synapomorphies: rudimentary or absence of anterior process of maxilla (5-1) and the anterior process being longer than the posterior process of pterygoid (49-1). Previously, crown Cryptobranchoidea were argued to have only two synapomorphies: fusion of the pubotibialis and puboischiotibialis, and fusion of hypobranchial I and ceratobranchial I (Estes, 1981). The first trait is obviously characteristic of cryptobranchoids considering that all salamandroids have these two muscles separate. However, our study indicates that fusion of the first branchial arch does not characterize *Regalerpeton* and several basal extant hynobiids (*Onychodactylus*, *Ranodon*, *Paradactyloson,* and *Pachyhynobius*) and is likely evolved in parallel in extant cryptobranchids and extant hynobiids. Both the total and crown group Cryptobranchoidea are each united by two other synapomorphies, including articular ossified (65-0) and postatlantal ribs unicapitate (84-1) for the former and the cultriform process of parasphenoid being anteriorly narrower bilaterally than or equal to the width of posteriorly (41-1) and opisthotic fused with exoccipital but with an independent prootic (56-1) for the latter.

### Limitations of the study

This study is limited by the few specimens known for *N. daohugouensis*: the newly referred specimen and the previously published details on the holotype specimen. As a result, the intraspecific variations found in this study for this taxon may need to be updated with more specimens yet to be discovered.

## STAR★Methods

### Key resources table

**Table:** 

REAGENT or Resource	SOURCE	IDENTIFIER
**Software and Algorithms**		

All figures were illustrated by using the Adobe Photoshop (version CC 19.1.6, X64) with Figure 1 created by an extension package, 3D Map Generator-Atlas, for Photoshop.	Adobe Inc.	RRID:SCR_014199; URL: https://www.adobe.com/products/photoshop.html
The Video S1 was created by using Adobe Premiere Pro CC (version 12.1.2).	Adobe Inc.	RRID:SCR_021315; URL: https://www.adobe.com/products/premiere.html
Micro-CT scanned data was processed by using the software VG Studio Max (version 2.2).	Volume Graphics	RRID:SCR_017997; URL: https://www.volumegraphics.com/de/produkte/vgstudio.html
Phylogenetic analyses were conducted by using the software T.N.T. (version 1.5).	T.N.T was initially described in Goloboff et al. (2003)	RRID:SCR_019122; URL: http://www.lillo.org.ar/phylogeny/tnt/

### Resource availability

#### Lead contact

Further information and requests for resources and reagents should be directed to and will be fulfilled by the lead contact, Jia Jia (jia_jia@pku.edu.cn).

#### Materials availability

The newly referred fossil specimen is reposited under the catalog number PKUP V0515 in the Peking University Paleontological Collections, Peking University, Beijing, China.

#### Data and code availability

The data matrix in nexus format used for cladistic analyses is available in the supplemental information.

### Experimental model and subject details

#### Animals

All salamander specimens used for comparative studies are either fossil specimens or extant specimens preserved in ethonal or formalin soluton curated in several institutions listed in Method Details.

### Method details

#### Specimens, developmental stage and life history

The specimen PKUP V0515 was collected by two of us (JJ and KQG) in 2018 from the Daohugou beds, cropping out at a quarry (41°18′32.0″N, 119°13′11.3″E) southeast of Xiayingzi as a unit of the Daohugou Village, approximately 1.7 km southwest of the Daohugou Palaeontological Fossil Museum (Figure 1). The specimen, cataloged as PKUP V0515 in the Peking University Paleontological Collections, is a nearly complete skeleton preserved with traces of soft tissues. Dorsoventrally compressed, the specimen is split and exposed on part and counterpart slabs of greyish-whitish mudstone (Figures 2 and 3). Referral of PKUP V0515 to *Neimengtriton daohugouensis* is justified by the following combination of features it shares with the holotype (IVPP V13393), but not with other stem hynobiids: snout broadly rounded; palatal process of pterygoid expanded bilaterally; vomer extended posteriorly, reaching close to the middle transverse plane of the cranium; vomerine tooth row located near posterior edge of vomer; cultriform process of parasphenoid anteroposteriorly shortened and anteriorly broadened; scapular blade shortened to slightly more than one-half width of coracoid; and metacarpals II and III not expanded anteroposteriorly.

PKUP V0515 has a snout-pelvic length (SPL) of 85.36 mm and a total length (TL) of 179.89 mm, and is notably larger than the holotype, which has an SPL of 75 mm and a TL “slightly greater than 140 mm” as reported by Wang (2004a: p. 858). We interpret the holotype IVPP V13393 of the species as a subadult (Jia and Gao, 2019), on the basis of its small body size and having only two carpals and three tarsals ossified in the mesopodium. In contrast, PKUP V0515 is an almost fully-grown adult, as evidenced by its substantially larger body size than the holotype and much more extensive ossification of the mesopodium displaying seven carpals in the manus and 11 tarsals in the pes. Our taxonomic revision of the genus and species as a new combination is primarily based on the information from PKUP V0515, supplemented by published accounts of other conspecific specimens (Wang, 2004a; Wang et al., 2008; Zhang et al., 2009; Sullivan et al., 2014). Our comparative study of fossil forms with extant hynobiids is largely based on published accounts (e.g., Gao et al., 1998; Gao and Shubin, 2001; Wang and Evans, 2006b; Zhang et al., 2009; Jia and Gao, 2016a; 2019; Rong, 2018), along with direct examination of specimens in various collections noted in supplemental information.

#### The age of the Daohugou beds

The Daohugou fossil beds are stratigraphically part of the Haifanggou Formation, correlative to the Jiulongshan Formation at the Western Hills in Beijing area (e.g., Chen et al., 2004; Gao et al., 2013; Ren, 2019); however, recent lithostratigraphic studies (Huang et al., 2015, 2018; Huang, 2016, 2019) show that the Haifanggou Formation at the Daohugou fossil beds are older than the Jiulongshan Formation in northern Hebei Province, the latter of which may represent phase transitions of the Upper Jurassic Tiaojishan Formation. The zircon U-Pb age of Haifanggou Formation at Beipiao is between 167.1 ± 0.9 Ma – 161.7 ± 1.9 Ma (Huang, 2019), and the age of the Daohugou beds has been dated at 166.7 ± 1.0 Ma (Chang et al., 2013) based on ^40^Ar/^39^Ar dating of rock samples from Beipiao area, and the overlapping beds of the Tiaojishan (Lanqi) Formation at the Daohugou section have been dated as 164–165 Ma (Chen et al., 2004; Liu et al., 2006; Yang and Li, 2008). This age determination is significant in relevance to our interpretation of the divergence time of the Hynobiidae from Cryptobranchidae, a profound cladogenetic event in the evolution of Cryptobranchoidea.

#### Preparation, CT scan, measurements, and terminology

The skeleton of PKUP V0515 was manually prepared using fine needles under a Nikon SMZ 745T microscope. After preparation, both slabs were photographed using a Nikon D90 camera and illustrated by using Adobe Photoshop^@^ CC (Adobe System Inc., San Jose, USA). As an aid to interpret the osteology of PKUP V0515, a Nikon XT H 320 LC micro-CT scanner at the Industrial Micro-CT Laboratory of China University of Geosciences, Beijing, China, was used to image selected regions of the skeleton. The craniocervical region in both slabs was scanned using the same current (60 μA) and voltage (175 kV) settings and, because no beam hardening artifacts were encountered, without any filtering. Scans along the longitudinal axis of the cranium yielded 16-bit tiff images, all with an image resolution of 2000 × 2000 pixels; slab A was imaged by 1999 slices at a voxel size of 16.64 μm, whereas slab B was imaged by 1753 slices at a voxel size of 20.00 μm. To reveal details of the right autopodium in the fore and hind limb, right femur, and pelvic girdle, the corresponding region in slab B was scanned at a current of 42 μA, voltage of 160 kV, and without any filtering. The resulting files (1999 slices of 16-bit tiff images) had a voxel size of 28.80 μm and an image resolution of 2000 × 2000 pixels. Scanning images of both the cranial and cervical regions were segmented and color rendered using the 3D software package VG Studio Max 2.2 (Volume Graphics, Heidelberg, Germany). For comparative studies, an adult specimen (ZMNH AA871) of the extant hynobiid *Hynobius amjiensis* was scanned (160 kV, 120 μA) using a same model micro-CT scanner at Zhejiang University in Hangzhou, with the resulting volume file having a voxel size of 42.89 μm.

Four linear measurements were measured for PKUP V0515 using hand-held calipers to the nearest 0.01 mm: total length (TL: distance along body axis between snout tip to posteriormost end of cartilaginous impression of tail); snout-pelvic length (SPL: distance along body axis between snout tip to posterior end of ischium); skull length (SKL: straight line distance between snout tip to posterior end of occipital condyles); and skull width (SKW: straight line distance across lateral edges of craniomandibular joints). We use double quotation marks around names of taxa that we regard as invalid (e.g., “*Liaoxitriton daohugouensis*”). Our anatomical terminology generally follows Francis (1934) and Trueb (1993), supplemented by terms used by Villa et al. (2014) for the braincase, Rose (2003) for the hyobranchial apparatus, and Shubin and Wake (2003) for the appendicular skeleton.

#### Phylogenetic analyses

A data matrix was constructed for the clade Cryptobranchoidea (Cryptobranchidae + Hynobiidae) that contains 26 characters recognized in this study based on our own observations on both fossil and extant specimens, and 82 characters from published data matrices (Zhao and Hu, 1984; Duellman and Trueb, 1986; Gao and Shubin, 2001, 2012; Jia and Gao, 2019). According to AmphibiaWeb (2021) and Frost (2021), Cryptobranchidae have two extant genera, including *Andrias* and *Cryptobranchus*; and Hynobiidae have nine extant genera, including *Batrachuperus*, *Hynobius*, *Liua*, *Onychodactylus*, *Pachyhynobius*, *Paradactylodon*, *Pseudohynobius*, *Ranodon,* and *Salamandrella*. Previously, the enigmatic hynobiid genus *Protohynobius* (Fei and Ye, 2000) was synonymized with the genus *Pseudohynobius* (Peng et al., 2010; Xiong et al., 2011); however, our recent study indicates that *Protohynobius* has multiple generic-level morphological differences from the latter (Jia et al., 2021), and therefore was retained here as an independent taxon. The data matrix contains 12 extant species in all 12 extant genera of Cryptobranchoidea, nine monotypic fossil genera from the Mesozoic strata of China (*Beiyanerpeton*, *Chunerpeton*, *Liaoxitriton*, *Linglongtriton*, *Neimengtriton*, *Nuominerpeton*, *Pangerpeton*, *Qinglongtriton*, *Regalerpeton*) and the Late Jurassic *Karaurus* as outgroup taxon. Fossil taxa of the crown Hynobiidae including *Parahynobius*, *Salamandrella* sp., *Ranodon* cf. *sibiricus* and Hynobiidae indet. and other Mesozoic taxa with purported relationships to Cryptobranchoidea were not included due to their incomplete preservation (e.g., *Iridotriton*, *Kiyatriton*) and lack of detailed morphological investigations (e.g., *Laccotriton*, *Sinerpeton*).

All taxa are coded based on published accounts mentioned in the main text and on the micro-CT scanned specimens as listed above. The cladistic analyses were undertaken in the software T.N.T version 1.5 (Goloboff and Catalano, 2016) with all characters set as equally weighted and unordered. In order to mitigate the impacts from neoteny, we followed previous studies (e.g., Wiens et al., 2005) to score question marks for the immature states of ontogeny-related characters (4, 33, 34, 37, 38, 44, 45, 47, 49, 50, 63, and 64; Data S1). A constrained heuristic search algorithm (100,000 replicates, 30 trees held per replication, tree bisection reconnection) was conducted using the cladogram of extant hynobiids built from 29 nuclear genes (Chen et al., 2015: figure 2A). Our first searches generated one most parsimonious tree (MPT; Figure 6) with a tree length of 203, a CI of 0.463 and a RI of 0.608. To test the relationship between modern species of Salamandroidea and the basal salamandroids *Beiyanerpeton* and *Qinglongtriton*, we included the modern ambystomatid *Ambystoma maculatum* into the original data matrix with codings based on Micro-CT scanned data of an adult specimen (FLMNH 26607) downloaded from the MorphoSource platform (https://www.morphosource.org/). Codings of *Ambystoma maculatum* are included in Data S1. Under the same analytical settings as in the first analysis, the second analysis generates 14 MPTs with a tree length of 234, a CI of 0.453 and a RI of 0.552. The strict consensus tree (Figure S5) of the 14 MPTs recovers a sister group relationship for *Ambystoma* and *Beiyanerpeton* and *Qinglongtriton*, but addition of this remote outgroup taxon (*Ambystoma*) brings no improvement in understanding interrelationships among species in the ingroup, the Cryptobranchoidea, as polymorphies appear at the base of Cryptobranchoidea and among stem hynobiids.

#### Caudosacral vertebrae definition and comparison

Caudosacral vertebrae are a series of postsacral vertebrae that are morphologically transitional between the more anterior trunk-sacral vertebrae and the more posterior caudal vertebrae. The caudosacrals were defined by Wake (1963) in plethodontids with the last caudosacral vertebra being the first postsacral vertebra to ventrally bear a fully developed haemal arch. In plethodontids, the haemal arch on the last caudosacral differs from those on the succeeding caudal vertebrae (1) in being strongly posteroventrally directed backwards, to the extent that its distal tip is well behind the level of the posterior end of the centrum, (2) its distal end is greatly broadened, and (3) it lacks the anteriorly directed, median hypophysial spine characteristic of caudal vertebrae. Similar patterns have been observed in the last caudosacrals in other living (Mivart, 1870; Jiang et al., 2018) and fossil (e.g., Jia and Gao, 2016a; 2016b; 2019) salamander taxa.

Such a definition of caudosacrals was overlooked by most previous studies on fossil salamanders, which instead identify caudosacrals as postsacral vertebrae with free ribs. Here, based on the definition of Wake (1963), we reinterpret caudosacral counts as three or four in *Nuominerpeton* and four in *Qinglongtriton*. The holotype of *Neimengtriton* was previously interpreted by us as having seven caudosacrals (Jia and Gao, 2019), but PKUP V0525 has only four. Based on published figures, we interpret that the holotypes of *Chunerpeton tianyiensis* (Gao and Shubin, 2003) and *Karaurus sharovi* (Ivachnenko, 1978) have four and five caudosacrals, respectively. Examination of an unpublished specimen (PKUP V0218) of *Pangerpeton sinensis* shows it has four caudosacrals(Gao et al., 2013). Interpretation of caudosacral counts for three other Chinese fossil salamanders are based on passages in the corresponding literature: two in *Jeholotriton*—“A haemal arch is present on the second caudal vertebra and more posterior ones” (Wang and Rose, 2005: p. 527); three in *Beiyanerpeton*—“The third and more posterior caudal vertebrae bear no free ribs, but dorsally carry a high neural spine and, ventrally, an elongate chevron” (Gao and Shubin, 2012: p. 5769); and four in *Regalerpeton*—“The first three caudosacrals bear free ribs. The remaining caudal vertebrae lack free ribs, but bear elongate transverse processes, and distinct neural and haemal arches” (Rong, 2018: p. 128). For *Valdotriton*, Evans and Milner (1996) reported three caudosacrals; based on their description and figure, we concur. Caudosacral counts are uncertain in the Chinese Early Cretaceous cryptobranchoids *Laccotriton*, *Liaoxitriton*, and *Sinerpeton* because the tail base region is not well preserved. In extant salamanders, a range of one to three caudosacrals is commonly seen among species of Hynobiidae (Xiong et al., 2013a) and may be typical for living urodeles in general (Mivart, 1870; Wake, 1966).

In temnospondyls, the postcranial skeletons often are not well preserved, and the caudosacrals are typically not differentiated from caudals. Usually, the postsacral vertebrae of temnospondyls and other early tetrapods are divided into anterior and posterior caudals, with the former referring to the first few caudals associated with ribs and lack haemal arches, and the latter referring to the remaining caudals that have haemal arches but lack ribs (e.g., Sigurdsen and Bolt, 2010). When we apply the criteria of Wake (1963) to temnospondyls and early tetrapods, the caudosacral counts equal to the number of anterior caudals plus one. The caudosacral counts in several temnospondyls (*Dendrerpeton acadianum*, *Doleserpeton annectens*, *Eryops megacephalus*, *Eoscopus lockardi*, *Mastodonsaurus giganteus*, *Siderops kehli*, *Archegosaurus decheni*) and early tetrapods (*Acanthostega gunnari*, *Ichthyostega stensioei*) are included here for comparison (Warren and Hutchinson, 1983; Coates, 1996; Jarvik, 1996; Holmes et al., 1998; Pawley, 2006; Witzmann and Schoch, 2006; Sigurdsen and Bolt, 2010; Clack, 2011).

#### Abbreviations

**Institutional abbreviations**—**CIB**, Chengdu Institute of Biology, Chinese Academy of Sciences, Chengdu, Sichuan Province, China; **CNU**, Capital Normal University, Beijing, China; **FLMNH**, Florida Museum of Natural History, Florida, USA; **FMNH**, Field Museum of Natural History, Chicago, Illionis, USA; **IVPP**, Institute of Vertebrate Paleontology and Paleoanthropology, Chinese Academy of Sciences, Beijing, China; **PKUP**, Peking University Paleontological Collections, Beijing, China; **ZMNH**, Zhejiang Museum of Natural History, Hangzhou, Zhejiang Province, China.

**Anatomical abbreviations**—**act**, acetabulum; **adf**, anterodorsal fenestra; **amf**, anteromedial fenestra; **an**, angular; **anf**, angular foramen; **app**, anterior process of prootic; **ar**, articular; **at**, atlas; **bb,** basibranchial; **bc**, basale commune; **bpp**, basal process of prootic; **c**, centrale; **cb,** ceratobranchial; **chn**, choana; **crd**, crista dorsalis of humerus; **crv**, crista ventralis of humerus; **dc**, distal carpal; **den**, dentary; **dg**, dentary groove; **dt**, distal tarsal; **f**, fibulare; **facf**, foramen faciale; **fe**, femur; **fi**, fibula; **fmn**, foramen mediale nasi; **fopt**, foramen palatinum; **fov**, fenestra ovalis; **fp**, foramen prooticum; **fpo**, foramen post-oticum; **fps**, footplate of stapes; **fr**, frontal; **hb**, hypobranchial; **hu**, humerus; **i**, intermedium; **idf**, inferior dental foramen; **il**, ilium; **isc**, ischium; **isp**, ischial spine; **lac**, lacrimal; **len**, lens; **mf**, mental foramen; **mp**, mental process; **mx**, maxilla; **na**, nasal; **obs**, orbitosphenoid; **opf**, optic foramen; **pa**, parietal; **pdp**, pars dorsalis of premaxilla; **pm**, premaxilla; **po**, postminimus; **pra**, prearticular; **prf**, prefrontal; **pro-op-exo**, prootic-opisthotic-exoccipital complex; **ps**, parasphenoid; **pt**, pterygoid; **qu**, quadrate; **ra**, radius; **rb**, rib; **sca**, scapulocoracoid; **sm**, septomaxilla; **sq**, squamosal; **ss**, stylus of stapes; **st**, stapes; **stf**, stapedial foramen; **t**, tibiale; **ti**, tibia; **tkv**, trunk vertebra; **tro**, trochanter; **u**, ulnare; **ul**, ulna; **vo**, vomer; **vpp**, ventral process of prootic; **y**, element y.

### Quantification and statitical analysis

This study does not use any statistical analysis.

## Supporting Citations

The following references appear in the supplemental information: Clemen and Greven, 2009; Dando et al., 2019; Edwards, 1976; Fei et al., 2006; Gaupp, 1911; Goodrich, 1930; Hecht and Edwards, 1977; Holmgren, 1933; Jurgens, 1971; Kingsbury and Reed, 1909; Lebedkina, 2004; Naylor, 1978; Parsons and Williams, 1962; Ryke, 1950; Sato, 1943; Shubin and Wake, 1996; Trueb and Cloutier, 1991; Vassilieva et al., 2015; Wilder, 1903; Xiong et al., 2013b; Zhang, 1985.

## Supplemental information

Document S1. Supplemental experimental procedures, Figures S1–S5, Table S1, and Data S1

## Footnotes

Supplemental information can be found online at https://doi.org/10.1016/j.isci.2021.102744.

## References

[bib1] AmphibiaWeb (2021). Information on Amphibian Biology and Conservation. http://amphibiaweb.org.

[bib2] Arntzen J.W., Beukema W., Galis F., Ivanović A. (2015). Vertebral number is highly evolvable in salamanders and newts (family Salamandridae) and variably associated with climatic parameters. Contrib. Zool..

[bib3] Averianov A.O., Tjutkova L.A. (1995). *Ranodon cf. sibiricus* (Amphibia, Caudata) from the Upper Pliocene of southern Kazakhstan: the first fossil record of the family Hynobiidae. Palaontol. Z..

[bib4] Blackburn D.C., Wake D.B., Zhang Z.-Q. (2011). Class Amphibia gray, 1825.

[bib5] Blaustein A.R., Johnson P.T. (2003). The complexity of deformed amphibians. Front. Ecol. Environ..

[bib6] Boisvert C.A. (2009). Vertebral development of modern sala manders provides insights into a unique event of their evolutionary history. J. Exp. Zool. B.

[bib7] Bonett R.M., Blair A.L. (2017). Evidence for complex life cycle constraints on salamander body form diversification. Proc. Natl. Acad. Sci. U S A.

[bib8] Chang S.-C., Zhang H., Hemming S.R., Meso G.T., Yang F. (2013). 40Ar/39Ar age constraints on the Haifanggou and Lanqi formations: when did the first flowers bloom?. Geol. Soc. Spec. Publ..

[bib9] Chen W., Ji Q., Liu D., Zhang Y., Song B., Liu X. (2004). [Isotope geochronology of the fossil-bearing beds in the Daohugou area, Ningcheng, Inner Mongolia]. Geol. Bull. China.

[bib10] Chen M.Y., Mao R.L., Liang D., Kuro-o M., Zeng X.M., Zhang P. (2015). A reinvestigation of phylogeny and divergence times of Hynobiidae (Amphibia, Caudata) based on 29 nuclear genes. Mol. Phylogenet. Evol..

[bib11] Clack J.A. (2011). A Carboniferous embolomere tail with supraneural radials. J. Vertebr. Paleontol..

[bib12] Clemen G., Greven H. (2009). Sex dimorphic dentition and notes on the skull and hyobranchium in the hynobiid salamander *Pachyhynobius shangchengensis* Fei, Qu & Wu, 1983 (Urodela: Amphibia). Vertebr. Zool..

[bib13] Coates M.I. (1996). The Devonian tetrapod *Acanthostega gunnari* Jarvik: postcranial anatomy, basal tetrapod interrelationships and patterns of skeletal evolution. Trans. R. Soc. Edinb..

[bib14] Dando M., Witzmann F., Kamenz S.K., Fröbisch N.B. (2019). How informative is vertebral development for the origin of lissamphibians?. J. Zool..

[bib15] de Queiroz K., Cantino P.D., Gauthier J.A. (2020). Phylonyms: A Companion to the PhyloCode.

[bib16] Dong F., Huang D.Y. (2011). A new elaterid from the middle jurassic Daohugou biota (Coleoptera: elateridae: protagrypninae). Acta Geol. Sin..

[bib17] Dong Z., Wang Y. (1998). A new urodele (*Liaoxitriton zhongjiani* gen. et sp. nov.) from the Early Cretaceous of western Liaoning Province, China. Vert. Palas..

[bib18] Duellman W.E., Trueb L. (1986). Biology of Amphibians.

[bib19] Duméril A.M.C. (1806). Zoologie Analytique, ou Méthode Naturelle de Classification des Animaux, Rendue Plus Facile à L’aide de Tableaux Synoptiques (Allais).

[bib20] Dunn E.R. (1922). The sound-transmitting apparatus of salamanders and the phylogeny of the Caudata. Am. Nat..

[bib21] Dunn E.R. (1923). The salamanders of the family Hynobiidae. Proc. Natl. Acad. Sci. U S A.

[bib22] Edwards J.L. (1976). Spinal nerves and their bearing on, salamander phylogeny. J. Morphol..

[bib23] Estes R. (1981). Encyclopedia of Paleoherpetology. Part 2, Gymnophiona, Caudata.

[bib24] Evans S.E., Milner A.L. (1996). A metamorphosed salamander from the early cretaceous of las hoyas, Spain. Philos. T. R. Soc. B.

[bib25] Evans S.E., Lally C., Chure D.C., Elder A., Maisano J.A. (2005). A late jurassic salamander (Amphibia: Caudata) from the morrison formation of north America. Zool. J. Linn. Soc..

[bib26] Fei L., Ye C. (2000). [A new hynobiid subfamily with a new genus and new species of Hynobiidae from west China. (Amphibia: Caudata)]. Cultum Herpetologica Sinica.

[bib27] Fei L., Hu S., Ye C., Huang Y. (2006).

[bib28] Fei L., Ye C. (2016).

[bib29] Francis E.T.B. (1934). The Anatomy of the Salamander.

[bib30] Frost D.R. (2021). Amphibian Species of the World: An Online Reference. http://research.amnh.org/herpetology/amphibia/index.html.

[bib31] Gao K.-Q., Cheng Z.W., Xu X. (1998). [First report of Mesozoic urodeles from China]. Chin. Geology..

[bib32] Gao K.-Q., Shubin N.H. (2001). Late Jurassic salamanders from northern China. Nature.

[bib33] Gao K.-Q., Shubin N.H. (2003). Earliest known crown-group salamanders. Nature.

[bib34] Gao K.-Q., Shubin N.H. (2012). Late jurassic salamandroid from western liaoning, China. Proc. Natl. Acad. Sci. U S A.

[bib35] Gao K.-Q., Chen J., Jia J. (2013). Taxonomic diversity, stratigraphic range, and exceptional preservation of Juro-Cretaceous salamanders from northern China. Can. J. Earth Sci..

[bib36] Gaupp E. (1911). Über den N. trochlearis der Urodelen und über die Austrittsstellen der Gehirnnerven aus dem Schädelraum im allgemeinen. Anat. Anz..

[bib37] Goloboff P.A., Farris J., Nixon K. (2003). TNT: tree analysis using new technology. Program Documentation.

[bib38] Goloboff P.A., Catalano S.A. (2016). TNT version 1.5, including a full implementation of phylogenetic morphometrics. Cladistics.

[bib39] Goodrich E.S. (1930). Studies on the Structure and Development of Vertebrates.

[bib40] Hanken J. (1984). Miniaturization and its effects on cranial morphology in plethodontid salamanders, genus *Thorius* (Amphibia: plethodontidae). I. Osteological variation. Biol. J. Linn. Soc..

[bib41] Hecht M.K., Edwards J.L., Hecht M.K., Goody P.C., Hecht B.M. (1977). The methodology of phylogenetic inference above the species level. Major Patterns in Vertebrate Evolution.

[bib42] Holmes R.B., Carroll R.L., Reisz R.R. (1998). The first articulated skeleton of *Dendrerpeton acadianum* (temnospondyli, dendrerpetontidae) from the lower pennsylvanian locality of joggins, nova scotia, and a review of its relationships. J. Vertebr. Paleontol..

[bib43] Holmgren N. (1933). On the origin of the tetrapod limb. Acta Zool..

[bib44] Houck J.D., Arnold S.J., Sever D.M. (2003). Courtship and mating behavior. Reproductive Biology and Phylogeny of Urodela.

[bib45] Huang D.Y. (2016). Daohugou Biota.

[bib46] Huang D.Y. (2019). Jurassic integrative stratigraphy and timescale of China. Sci. China Earth Sci..

[bib47] Huang D.Y., Cai C., Jiang J., Su Y., Liao H. (2015). Daohugou bed and fossil record of its basal conglomerate section. Acta Palaeontologica Sinica.

[bib48] Huang D.Y., Cai C., Fu Y.Z., Su Y.T. (2018). The middle-late jurassic Yanliao entomofauna. Palaeoentomology.

[bib49] Ivachnenko M.F. (1978). Urodelans from the triassic and jurassic of soviet central Asia. Paleontol. J..

[bib50] Jarvik E. (1996). The Devonian Tetrapod *Ichthyostega*.

[bib51] Jia J., Gao K.-Q. (2016). A new hynobiid-like salamander (Amphibia, Urodela) from Inner Mongolia, China, provides a rare case study of developmental features in an Early Cretaceous fossil urodele. PeerJ.

[bib52] Jia J., Gao K.-Q. (2016). A new basal salamandroid (Amphibia, Urodela) from the late jurassic of qinglong, Hebei province, China. PLoS One.

[bib53] Jia J., Gao K.-Q. (2019). A new stem hynobiid salamander (Urodela, Cryptobranchoidea) from the upper jurassic (oxfordian) of liaoning province, China. J. Vertebr. Paleontol..

[bib54] Jia J., Jiang J., Zhang M., Gao K.-Q. (2019). Osteology of *Batrachuperus yenyuanensis* (Urodela, Hynobiidae), a high-altitude mountain stream salamander from western China. PLoS One.

[bib55] Jia J., Gao K.-Q., Jiang J., Bever G., Xiong R., Wei G. (2021). Comparative osteology of the hynobiid complex *Liua-Protohynobius-Pseudohynobius* (Amphibia, Urodela): I. Cranial anatomy of *Pseudohynobius*. J. Anat..

[bib56] Jiang J., Jia J., Zhang M., Gao K.-Q. (2018). Osteology of *Batrachuperus londongensis* (Urodela, Hynobiidae): study of bony anatomy of a facultatively neotenic salamander from mount emei, sichuan province, China. PeerJ.

[bib57] Jurgens J.D. (1971). The morphology of the nasal region of Amphibia and its bearing on the phylogeny of the group. Annale Universiteit Van Stellenbosch.

[bib58] Kingsbury B.F., Reed H.D. (1909). The columella auris in Amphibia. Second Contribution. J. Morphol..

[bib59] Kuzmin S.L., Thiesmeier B. (2001). Mountain Salamanders of the Genus *Ranodon*.

[bib60] Larson A., Weisrock D.W., Kozak K.H., Sever D.M. (2003). Phylogenetic systematics of salamanders (Amphibia: Urodela), a review. Reproductive Biology and Phylogeny of Urodela (Amphibia).

[bib61] Lebedkina N.S. (2004). Evolution of the Amphibian Skull.

[bib62] Ledbetter N.M., Bonett R.M. (2019). Terrestriality constrains salamander limb diversification: implications for the evolution of pentadactyly. J. Evol. Biol..

[bib63] Linnaeus C. (1758). Systema Naturae Per Regna Tria Naturae, Secundum Classes, Ordines, Genera, Species, Cum Characteribus, Differentiis, Synonymis, Locis. Tomus I.

[bib64] Litvinchuk S.N., Borkin L.J. (2003). Variation in number of trunk vertebrae and in count of costal grooves in salamanders of the family Hynobiidae. Contrib. Zool..

[bib65] Liu Y., Liu Y., Ji S., Yang Z. (2006). U-Pb zircon age for the Daohugou Biota at Ningcheng of Inner Mongolia and comments on related issues. Sci. Bull..

[bib66] Luo Q., Tong F., Song Y., Wang H., Du M., Ji H. (2018). Observation of the breeding behavior of the Chinese Giant Salamander (*Andrias davidianus*) using a digital monitoring system. Animals.

[bib67] Mivart G. (1870). On the axial skeleton of the Urodela. Proc. Zool. Soc. Lond..

[bib68] Naylor B.G. (1978). The Systematics of Fossil and Extant Salamanders (Amphibia, Caudata); with Special Reference to the Vertebral Column and Trunk Musculature.

[bib69] Noble G.K. (1931). The Biology of the Amphibia.

[bib70] Parsons T.S., Williams E.E. (1962). The teeth of Amphibia and their relation to amphibian phylogeny. J. Morphol..

[bib71] Pawley K. (2006). The Postcranial Skeleton of Temnospondyls (Tetrapoda: Temnospondyli).

[bib72] Peng R., Zhang P., Xiong J.-L., Gu H.-J., Zeng X.-M., Zou F.-D. (2010). Rediscovery of *Protohynobius puxiongensis* (Caudata, Hynobiidae) and its phylogenetic position based on complete mitochondrial genomes. Mol. Phylogenet. Evol..

[bib73] Poyarkov N.A., Che J., Min M.-S., Kuro-o M., Yan F., Li C., Iizuka K., Vieites D. (2012). Review of the systematics, morphology and distribution of Asian Clawed Salamanders, genus *Onychodactylus* (Amphibia, Caudata: Hynobiidae), with the description of four new species. Zootaxa.

[bib74] Pyron R.A., Wiens J.J. (2011). A large-scale phylogeny of Amphibia including over 2800 species, and a revised classification of extant frogs, salamanders, and caecilians. Mol. Phylogenet. Evol..

[bib75] Ratnikov V.Y. (2010). A review of tailed amphibian remains from late Cenozoic sediments of the East European plain. Russ. J. Herpetol..

[bib76] Regal P.J. (1966). Feeding specializations and the classification of terrestrial salamanders. Evolution.

[bib77] Ren D., Ren D., Shih C.K., Gao T., Yao Y., Wang Y. (2019). Jurassic-Cretaceous non-marine stratigraphy and entomogaunas in northern China. Rhythms of Insect Evolution.

[bib78] Rong Y.F. (2018). Restudy of *Regalerpeton weichangensis* (Amphibia: Urodela) from the lower cretaceous of Hebei, China. Vert. PalAs.

[bib79] Rose C.S., Heatwole H., Davies M. (2003). The developmental morphology of salamander skulls. Amphibian Biology, Volume 5: Osteology.

[bib80] Ryke P.A.J. (1950). Contributions to the cranial morphology of the Asiatic urodele *Onychodactylus japonicus* Houttuijn. Ann. Univ. Stellenbosch.

[bib81] Sato I. (1943). A Monograph of the Tailed Batrachians of Japan (Nippon Shuppan-Sha).

[bib82] Schmalhausen I.I. (1968). The Origin of Terrestrial Vertebrates.

[bib83] Sever D.M. (1991). Comparative anatomy and phylogeny of the cloacae of salamanders (Amphibia: Caudata). I. Evolution at the family level. Herpetologica.

[bib84] Shubin N.H., Wake D.B., Crawford A.J. (1995). Morphological variation in the limbs of *Taricha granulosa* (Caudata: salamandridae): evolutionary and phylogenetic implications. Evolution.

[bib85] Shubin N.H., Wake D.B. (1996). Phylogeny, variation, and morphological integration. Am. Zool..

[bib86] Shubin N.H., Wake D.B., Heatwole H., Davies M. (2003). Morphological variation, development, and evolution of the limb skeleton of salamanders. Amphibia Biology, Volume 5: Osteology.

[bib87] Sigurdsen T., Bolt J.R. (2010). The Lower Permian amphibamid *Doleserpeton* (Temnospondyli: dissorophoidea), the interrelationships of amphibamids, and the origin of modern amphibians. J. Vertebr. Paleontol..

[bib88] Skutschas P.P. (2014). *Kiyatriton leshchinskiyi* Averianov et Voronkevich, 2001, a crown-group salamander from the Lower Cretaceous of Western Siberia, Russia. Cretac. Res..

[bib89] Skutschas P.P. (2016). A new crown-group salamander from the middle jurassic of western siberia, Russia. Palaeobio. Palaeoenv..

[bib90] Sullivan C., Wang Y., Hone D.W.E., Wang Y., Xu X., Zhang F. (2014). The vertebrates of the jurassic Daohugou biota of northeastern China. J. Vertebr. Paleontol..

[bib91] Syromyatnikova E.V. (2014). The first record of *Salamandrella* (Caudata: Hynobiidae) from the Neogene of Russia. Russ. J. Herpetol..

[bib92] Trueb L., Hanken J., Hall B.K. (1993). Patterns of cranial diversity among the Lissamphibia. The Skull, Volume 2: Patterns of Structural and Systematic Diversity.

[bib93] Trueb L., Cloutier R., Schultze H.P., Trueb L. (1991). A phylogenetic investigation into the inter- and intrarelationships of the Lissamphibia (Amphibia: temnospondyli). Origins of the Higher Groups of Tetrapods: Controversy and Consensus.

[bib94] Vasilyan D., Böhme M., Chkhikvadze V.M., Semenov Y.A., Joyce W.G. (2013). A new giant salamander (Urodela, pancryptobrancha) from the Miocene of eastern Europe (grytsiv, Ukraine). J. Vertebr. Paleontol..

[bib95] Vasilyan D., Zazhigin V.S., Böhme M. (2017). Neogene amphibians and reptiles (Caudata, Anura, gekkota, lacertilia, and Testudines) from the south of western siberia, Russia, and northeastern Kazakhstan. PeerJ.

[bib96] Vassilieva A.B., Lai J., Yang S., Chang Y., Poyarkov N.A. (2015). Development of the bony skeleton in the Taiwan salamander, *Hynobius formosanus* Maki, 1922 (Caudata: Hynobiidae): heterochronies and reductions. Vertebr. Zool..

[bib97] Venczel M. (1999). Fossil land salamanders (Caudata, Hynobiidae) from the Carpathian basin: relation between extinct and extant genera. Acta Palaeontol. Rom..

[bib98] Venczel M. (1999). Land salamanders of the family Hynobiidae from the neogene and quaternary of Europe. Amphib. Reptil..

[bib99] Venczel M., Hír J. (2013). Amphibians and squamates from the Miocene of felsötárkány basin, N-Hungary. Palaeontogr. Abt. A.

[bib100] Villa A., Andreone F., Boistel R., Delfino M., Corti C., Capula M. (2014). Skull and lower jaw osteology of the Lanza’s salamander, *Salamandra lanzai* (Amphibia, Caudata). Scripta Herpetologica. Studies on Amphibians and Reptiles in Honour of Benedetto Lanza.

[bib101] Vitt L.J., Caldwell J.P. (2014). Herpetology: An Introductory Biology of Amphibians and Reptiles.

[bib102] Wake D.B. (1963). Comparative osteology of the plethodontid salamander genus *Aneides*. J. Morphol..

[bib103] Wake D.B. (1966). Comparative osteology and evolution of the lungless salamanders, family Plethodontidae. Mem. South. Calif. Acad. Sci..

[bib104] Wake D.B., Dresner I.G. (1967). Functional morphology and evolution of tail autotomy in salamanders. J. Morphol..

[bib105] Wake D.B. (2009). What salamanders have taught us about evolution. Annu. Rev. Ecol. Evol. Syst..

[bib106] Wang Y. (2000). A new salamander (Amphibia: Caudata) from the early cretaceous Jehol biota. Vert. Palas..

[bib107] Wang Y. (2004). A new Mesozoic caudate (*Liaoxitriton daohugouensis* sp. nov.) from Inner Mongolia. China. Sci. Bull..

[bib108] Wang Y. (2004). Taxonomy and stratigraphy of Late Mesozoic anurans and urodeles from China. Acta Geol. Sin..

[bib109] Wang Y., Rose C.S. (2005). *Jeholotriton paradoxus* (Amphibia: Caudata) from the lower cretaceous of southeastern inner Mongolia, China. J. Vertebr. Paleontol..

[bib110] Wang Y., Evans S.E. (2006). Advances in the study of fossil amphibians and squamates from China: the past fifteen years. Vert. Palas..

[bib111] Wang Y., Evans S.E. (2006). A new short-bodied salamander from the Upper Jurassic/Lower Cretaceous of China. Acta Palaeontol. Pol..

[bib112] Wang Y., Zhang G., Sun A., Li J., Wu X., Zhang F. (2008). Tetrapoda. The Chinese Fossil Reptiles and Their Kin.

[bib113] Wang Y., Dong L., Evans S.E. (2016). Polydactyly and other limb abnormalities in the Jurassic salamander *Chunerpeton* from China. Palaeobiodivers. Palaeoenviron..

[bib114] Warren A.A., Hutchinson M.N. (1983). The last labyrinthodont? A new brachyopoid (Amphibia, temnospondyli) from the early jurassic evergreen formation of queensland, Australia. Philos. T. R. Soc. B.

[bib115] Weisrock D.W., Macey J.R., Matsui M., Mulcahy D.G., Papenfuss T.J. (2013). Molecular phylogenetic reconstruction of the endemic Asian salamander family Hynobiidae (Amphibia, Caudata). Zootaxa.

[bib116] Wiens J.J., Bonettm R.M., Chippindale P.T. (2005). Ontogeny discombobulates phylogeny: paedomorphosis and higher-level salamander relationships. Syst. Biol..

[bib117] Wilder H.H. (1903). The skeletal system of *Necturus maculatus* Rafinesque. Mem. Read. Boston Soc. Nat. Hist..

[bib118] Witzmann F., Schoch R.R. (2006). The postcranium of *Archegosaurus decheni*, and a phylogenetic analysis of temnospondyl postcrania. Palaeontology.

[bib119] Xiong J.-L., Gu H.-J., Gong T.-J., Zeng X.-M. (2011). Redescription of an enigmatic salamander, *Pseudohynobius puxiongensis* (Fei et Ye, 2000) (Urodela: Hynobiidae). Zootaxa.

[bib120] Xiong J.-L., Yu P.-J., Zhang J.-L., Zhu W.-W., Sun P. (2013). [Vertebral column characteristics of *Batrachuperus pinchonii*, and discussion on the division of the vertebral column in Urodela]. Chin. J. Zool..

[bib121] Xiong J.-L., Sun P., Zhang J.-L., Liu X.-Y. (2013). A comparative study of the hyobranchial apparatus in Hynobiidae (Amphibia: Urodela). Zoology.

[bib122] Yang W., Li S. (2008). Geochronology and geochemistry of the Mesozoic volcanic rocks in Western Liaoning: implications for lithospheric thinning of the North China Craton. Lithos.

[bib123] Zhang F. (1985). On anatomy of the skeletal system of *Liua shihi* (Liu) (Amphibia: Hynobiidae). Acta Herpetol. Sin..

[bib124] Zhang L.J., Fan G.Q. (2001). *Voldotriton* [sic] *sinensis* sp. nov.–a new species of Mesozoic salmander [sic]. Land Resour..

[bib125] Zhang P., Chen Y.-Q., Zhou H., Liu Y.-F., Wang X.-L., Papenfuss T.J., Wake D.B., Qu L.-H. (2006). Phylogeny, evolution, and biogeography of Asiatic salamanders (Hynobiidae). Proc. Natl. Acad. Sci. U S A.

[bib126] Zhang P., Wake D.B. (2009). Higher-level salamander relationships and divergence dates inferred from complete mitochondrial genomes. Mol. Phylogenet. Evol..

[bib127] Zhang G., Wang Y., Jones M.E.H., Evans S.E. (2009). A new Early Cretaceous salamander (*Regalerpeton weichangensis* gen. et sp. nov.) from the Huajiying Formation of northeastern China. Cretac. Res..

[bib128] Zhao E.-M., Hu Q.X. (1984). Studies on Chinese Tailed Amphibians.

[bib129] Zhou Z.-H., Wang Y. (2017). Vertebrate assemblages of the jurassic Yanliao biota and the early cretaceous Jehol biota: comparisons and implications. Palaeoworld.

