# Supplementary material for: Middle Jurassic stem hynobiids from China shed light on the evolution of basal salamanders
Source: iScience. 2021 Jun 17;24(7):102744. doi: 10.1016/j.isci.2021.102744 (PMC8264161; doi:10.1016/j.isci.2021.102744)
Supplement: Document S1. Supplemental experimental procedures, Figures S1–S5, Table S1, and Data S1 [file mmc1.pdf]

iScience, Volume *24*

Supplemental information

Middle Jurassic stem hynobiids from China shed light on the evolution of basal salamanders

Jia Jia, Jason S. Anderson, and Ke-Qin Gao

1 Description of *Neimengtriton daohugouensis* comb. nov., Related to STAR Methods

General remarks

PKUP V0515 is informative both for revealing details in the dermal skull roof, suspensorium, braincase, mandible, autopodium, and tail of *Neimengtriton daohugouensis* that are unknown or poorly known from the holotype and, in combination with the holotype, for revealing intraspecific variation in caudosacral vertebral counts and in manual and pedal phalangeal formulae. Traces of soft tissue preserved in PKUP V0515 further reveal the presence of at least nine costal grooves along the trunk and a moderately prominent dorsal fin along the tail (see Discussion). The tip of the tail is missing in the holotype, but in PKUP V0515 the tail is complete and longer (94.53 mm in length) than its SPL. With a maximum known TL of nearly 180 mm, *N. daohugouensis* falls midway within the size range of living hynobiids, whose adult TLs range from 70–270 mm (Fei and Ye, 2016), and is larger than other currently recognized stem hynobiids, whose maximum known or estimated TLs range from 140–166.52 mm, as follows: 140 mm for *Liaoxitriton* (Dong and Wang, 1998); 158. 08 mm for *Linglongtriton* (Jia and Gao, 2019); 166.52 mm for *Nuominerpeton* (Jia and Gao, 2016a); and at least 146 mm for *Regalerpeton* (estimated from Rong, 2018: figures 1, 2). Whereas the skull in the holotype of *N. daohugouensis* appears undistorted and is wider than long (SKW = 21 mm vs. SKL = 19 mm in Wang, 2004a), the skull in PKUP V0515 has suffered slight taphonomic distortion obliquely across its posterior portion, resulting in its anatomical left side being pushed inwards (Figures S1, S2). Consequently, the preserved skull in PKUP V0515 is slightly narrower than long (SKW = 18.94 mm vs. SKL = 19.63 mm). Both PKUP V0515 and the holotype are interpreted as postmetamorphic because each lacks both internal and external gills (i.e., no evidence of gill rakers or filaments) and their pterygoids bear a palatal process that is short and directed anterolaterally towards to the posterior end of the maxilla, typical features for extant metamorphosed hynobiids (Fei and Ye, 2016).

Skull

Dermal skull roof—The skull bones are unsculptured dorsally both in PKUP V0515 and

the holotype, a condition that is typical for both living and fossil panhynobians (e.g., Clemen and Greven, 2009; Jiang et al., 2018; Rong, 2018).

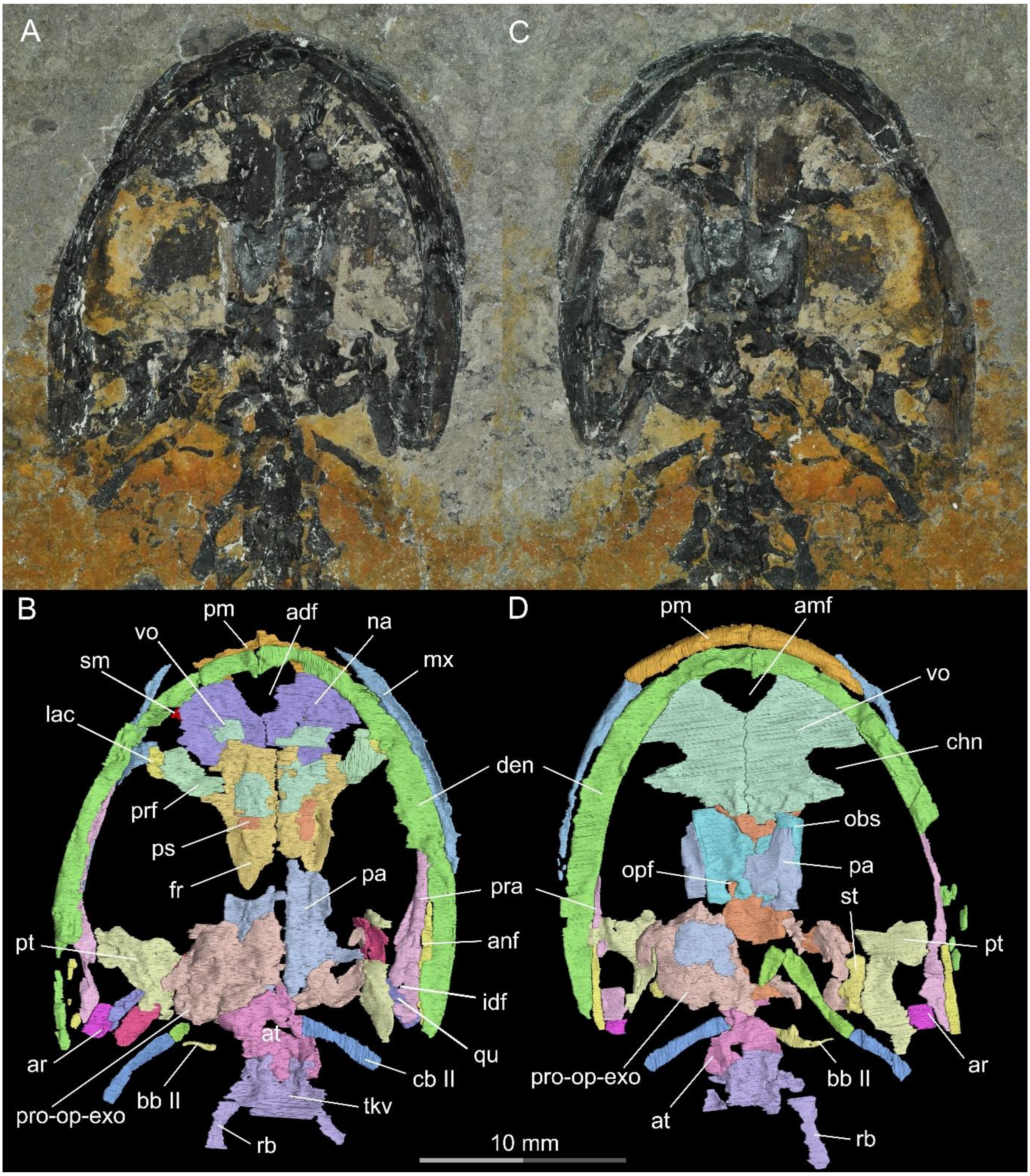

Figure S1. Photographs and micro-CT rendered reconstructions of craniocervical region of the newly referred specimen (PKUP V0515) of the stem hynobiid *Neimengtriton daohugouensis* comb. nov., Related to Figures 2, 3: (A) photograph of bones exposed at surface and (B) color-coded reconstruction of exposed and embedded bones, both as preserved in slab A; and (C) photograph of bones exposed at surface and (D) color-coded

reconstruction of exposed and embedded bones, both as preserved in slab B. See STAR Methods for abbreviations.

The premaxillae (Figures S1, S2) are paired, contacting each other medially and the maxilla laterally. The premaxilla is slightly shorter mediolaterally than one-half the length of the maxilla, as in *Pangerpeton* and most panhynobians, except *Regalerpeton*, the latter being a neotenic taxon with its maxilla shortened to the length of its premaxilla (Rong, 2018: figure 5A, 5B). The pars dorsalis and part of the pars palatina of both premaxillae are preserved on slab A of PKUP V0515 (Figures S1A, S1B, S2A), with the remaining parts of the pars palatina and both partes dentalis preserved on slab B (Figures S1C, S1D, S2B). The pars dorsalis (alary process) of the premaxilla is moderately elongate dorsally and spike-like, with its tapering dorsal apex overlapping the nasal as in *Pangerpeton* and other stem hynobiids. The posterior end of the pars dorsalis terminates at the anteroposterior midpoint of the nasal and fails to reach the frontal (Figure S2A). The paired premaxillary partes dorsalis are separated from each other by the anterodorsal fenestra and the anteromedian portions of the paired nasals (Figure S2A). The pars palatina of the premaxilla is a narrow bony ledge extending along the entire medial aspect of the premaxilla (Figure S2B). The premaxillary partes palatina are in contact medially with each other and laterally with the pars palatina of the adjacent maxilla. Where the partes palatina of the premaxilla and maxilla meet, they form the bony floor of the external naris. Although dorsoventral compression of the skull in PKUP V0515 has displaced both premaxillae anteriorly from the vomers, in life the pars palatina of each premaxilla would have contributed to the palate by articulating posteriorly with the vomer. The pars dentalis of the premaxilla is shallow and medially bore a row of small and closely packed teeth (see dentition account, below).

The anterodorsal fenestra (Figures S1B, S2A) is a median opening bounded anteriorly by the premaxillae and posteriorly by the nasals. The fenestra is spindle shaped in dorsal view, being constricted at both its anterior and posterior ends, and with the width across its middle part approximately one-half the width of either external naris (width of latter measured with skull re-positioned obliquely, with the external naris facing directly towards the viewer). A similar-sized anterodorsal fenestra is present in *Pangerpeton* and most panhynobians, whereas

the fenestra is narrowed to varying extents in some living hynobiids (e.g., *Pachyhynobius*; Clemen and Greven, 2009) and is closed (absent) in some species of *Hynobius* and *Salamandrella* (Sato, 1943; Fei et al., 2006) as a derived feature within Hynobiidae.

The nasals (Figures S1B, S2A) are irregular in outline, slightly longer than wide, and posteriorly contact each other along a straight median suture. The paired nasals are anteromedially separated from each other by the anterodorsal fenestra. The nasals are slightly wider than the frontals, a plesiomorphic feature of urodeles (Gao and Shubin, 2012). The nasal overlaps the frontal posteriorly and, in life, would have articulated laterally with the prefrontal and maxilla, although these bones in PKUP V0515 are displaced postmortem and separated from one another. The intervening prefrontal ensures that the nasal remains free from contacting the lacrimal (see below). The anterior border of the nasal is smooth as in *Pangerpeton* and other stem hynobiids, whereas the nasal is anteriorly bifurcated by a triangular notch for receipt of the premaxillary pars dorsalis in living hynobiids (e.g., *Hynobius* of Vassilieva et al., 2015; *Batrachuperus* of Jiang et al., 2018; Jia et al., 2019), a feature we deem diagnostic for crown Hynobiidae. In PKUP V0515, the middle portion of the left nasal is penetrated by a small foramen, the foramen mediale nasi (Figure S2A), for the passage of distal branches of the superficial ophthalmic nerve (cranial nerve [CN] V_1) and associated veins supplying the skin of the snout as in living salamanders (Jurgens, 1971).

The frontals (Figures 2, S1, S2A) are elongate bony plates, broadest anteriorly, and with their posterior ends slightly surpassing the anteroposterior midpoint of the orbit. Each frontal articulates with its counterpart medially along a straight suture, and is overlapped anteriorly by the nasal and anterolaterally by the prefrontal. CT images of PKUP V0515 reveal that the frontals are convex dorsally and concave ventrally, even though the skull roof has been dorsoventrally compressed. The lateral border of the frontal curves ventrally to articulate with the orbitosphenoid and the anterolateral process of the parietal, and contributes to less than one-third the length of the medial border of the orbit, which is completed by the prefrontal anteriorly and the parietal posteriorly. The posterior process of the frontal is triangular and posteriorly pointed in outline, and overlaps the parietal as indicated by the impression of the anterior part of the right parietal on the ventral surface of the frontal on slab A (Figures 2B, S1B; see below). The paired posterior processes of the frontals diverge from each other. The

tiny median opening between the posterior ends of the frontals and the anterior ends of the parietals is the frontoparietal fontanelle (Figure 2B). Among other stem hynobiids, the frontoparietal fontanelle is only present in *Regalerpeton* (Rong, 2018).

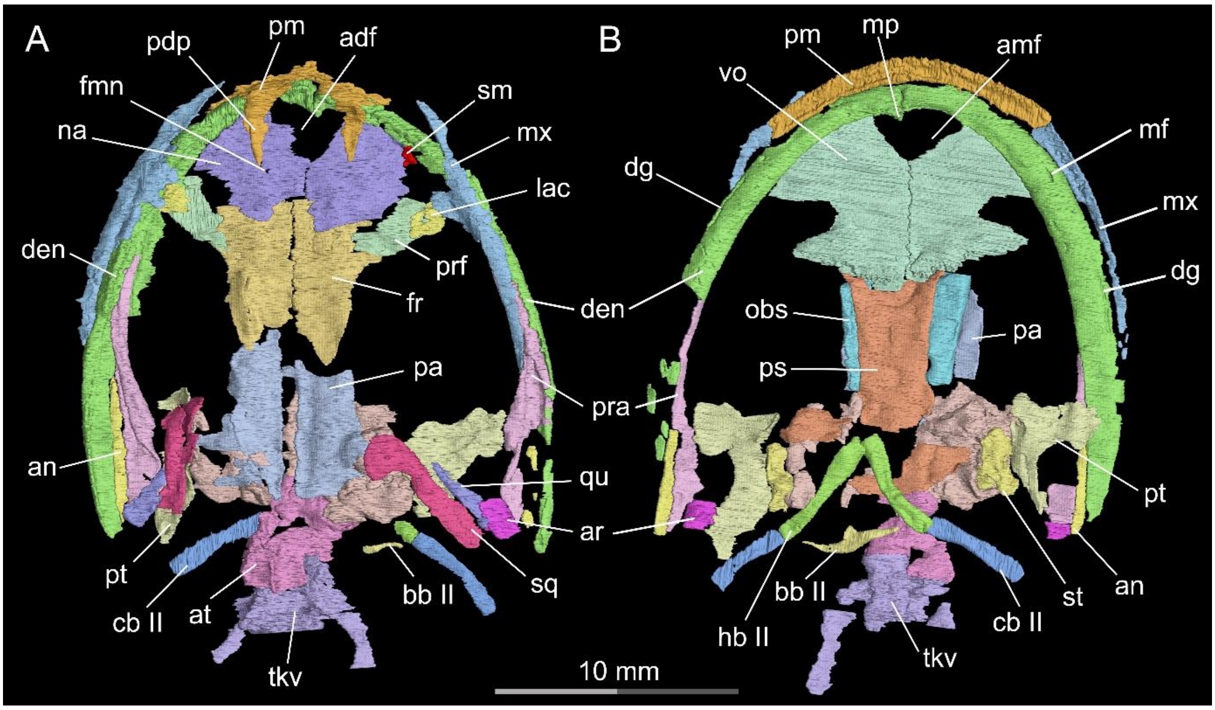

Figure S2. Micro-CT rendered reconstructions of the entire craniocervical region of the newly referred specimen (PKUP V0515) of the stem hynobiid *Neimengtriton daohugouensis* comb. nov., based on bones preserved in both slabs A and B, Related to Figures 2, 3. (A) dorsal and (B) ventral views. See STAR Methods for abbreviations.

The parietals (Figures 2, 3, S1, S2) are paired, and somewhat boot-like in dorsal or ventral outline, with the "toe" formed by the stout posterolateral process. Similar to the frontals, the paired parietals have a straight articulation with one another along the skull midline and the anterior portion of the lateral border curves downward to contact the orbitosphenoid. Each parietal is about 30% longer than its corresponding frontal, thanks to the anterolateral process of the parietal extending anteriorly nearly to the level of the anterior border of orbitosphenoid (Figures S1B, S1D, S2B); we regard elongate parietals as an autopomorphy for *Neimengtriton daohugouensis*. The anterolateral process of the parietal is

triangular and laterally short, wedging between the frontal and the orbitosphenoid. Posterior to the anterolateral process, the quadrate process of the parietal (Wilder, 1903) is short and extends ventrolaterally; its ventral end may have articulated with the cartilaginous ascending process of the palatoquadrate as in living hynobiids (e.g., Ryke, 1950; Lebedkina, 2004; Jiang et al., 2018). The base of the quadrate process is not penetrated by a foramen, indicating that the trochlear nerve (CN IV; Gaupp, 1911) may have exited the braincase by passing through the fissure between the parietal and the orbitosphenoid as in *Salamandra* (Francis, 1934). The posterolateral process of the parietal is short, stout, and directed laterally where it overlaps the otic complex and its lateral end articulates with the squamosal. The posterior process of the parietal is short and overlaps the otic complex.

The maxilla (Figures 2, 3, S1, S2) is elongate, extending from below the anteroposterior midpoint of the nasal posteriorly to slightly past the mid-level of the orbit. The anterior process of the maxilla is elongate, with a tapered distal end overlapping the premaxilla as in *Pangerpeton* and other stem hynobiids (e.g., Jia and Gao, 2019). The pars facialis (dorsal process) of the maxilla is trapezoidal in lateral view (Figure S2A), extending along the dorsal border of the maxilla, and articulating dorsally with the nasal and lacrimal, thereby preventing the lacrimal from entering the border of the external naris (see below). The pars palatina, as best shown in slab B (Figure S2B) is a narrow bony ledge extending along the medial surface of the maxilla. Similar to the premaxilla, the pars palatina of the maxilla would have articulated with the vomer when the individual was alive, but due to postmortem displacement, both maxillae have been shifted laterally. The pars dentalis of the maxilla bears a row of small teeth, extending from its contact with the premaxilla to the posterior extremity of the posterior process. The posterior process of the maxilla is elongate and lacks any bony contact medially with the palatal process of the pterygoid.

The septomaxilla is not preserved in the left side of the skull, but is well preserved on the right side in slab A (Figures S1B, S2A). The septomaxilla is dislocated postmortem to the anterior border of the external naris; in life, it would have been located at the posteroventral corner of the external naris, where it contributes to the passage of the nasolacrimal duct as in living hynobiids (e.g., Jiang et al., 2018). CT images reveal that the septomaxilla is small, irregular, anteriorly constricted, and posteriorly expanded as commonly seen in hynobiids

(e.g., Jiang et al., 2018).

The lacrimal (Figures S1B, S2A) is roughly “L”-shaped, with a short medial process overlapping the prefrontal. The lacrimal is flat and penetrated by a large foramen in its middle part (Figure S2A) for passage of the nasolacrimal duct. The lacrimal laterally articulates with the pars facialis of the maxilla and medially with the prefrontal, the latter of which prevents the lacrimal from contacting the nasal. As a result, the lacrimal contributes to the anterior border of the orbit, but does not enter the external naris anteriorly; a similar pattern also occurs in the stem hynobiid *Nuominerpeton* (Jia and Gao, 2016a). By contrast, in the stem hynobiid *Linglongtriton*, the lacrimal enters only into the external naris (Jia and Gao, 2019); that is a plesiomorphic feature of panhynobian, because it is present in the stem urodele *Karaurus* (Ivachnenko, 1978). The lacrimal enters into both the naris and orbit in *Regalerpeton* (Rong, 2018), whereas conditions remain unknown in *Liaoxitriton* and *Pangerpeton*. In crown hynobiids, the lacrimal has four patterns with regard to its contribution to the orbital rim and external naris (see Jia and Gao, 2016a; 2019).

The prefrontals (Figures S1B, S2A) are paired, mediolaterally elongate, and positioned obliquely along the anterodorsal border of the orbit. The prefrontal is expanded laterally and constricted medially. The prefrontal articulates with the frontal medially and the lacrimal laterally, but is separated from the maxilla by the latter bone.

Suspensorium—The squamosal (Figure S2A) is roughly “L”-shaped, with a moderately expanded proximal end and an elongate distal ramus. Due to postmortem deformation of the skull in PKUP V0515, the squamosal as best seen on the right side of the skull has been rotated clockwise and now lies obliquely in an anteromedial-posterolateral orientation spanning between the parietal and the jaw joint; in life, the squamosal would have extended ventrolaterally and perpendicular to the anteroposterior axis of the skull. The proximal head of the squamosal is rounded, with a convex proximal border that remains in articulation with the posterolateral process of the parietal (Figure S2A). The otic process (anterior process) of the squamosal is absent as in most other stem hynobiids, yet occurs as a short, blunt process in the neotenic *Regalerpeton* (Rong, 2018). Extending posteriorly from the expanded proximal end of the bone, the posterior process of the squamosal is short, stout, and projects at a right angle from the long axis of the squamosal as in living salamanders. The bony ramus

of the squamosal is elongate, dorsally convex and ventrally concave, and slightly expanded distally for overlapping the quadrate (Figure S2A).

The quadrate (Figures S1B, S2A) is elongate, being slightly longer than one-half the length of the squamosal, and is preserved articulating dorsally with the squamosal and ventrally with the pterygoid and articular. The quadrate tapers proximally and is expanded distally as commonly seen in panhynobians; however, our recent studies (e.g., Jiang et al., 2018; Jia et al., 2019) found that the distal end of the quadrate in living hynobiids is proportionally more expanded along the sagittal axis of the skull than in stem hynobiids (e.g., Jia and Gao, 2019), a feature needs to be scrutinized in future studies. Similar to the squamosal, CT images reveal that the quadrate is convex dorsally and concave ventrally.

The pterygoid (Figures S1B, S1D, S2) is triradiate, consisting of an anterolaterally directed palatal process, a dorsomedially directed otic process, and a posterolaterally directed quadrate process. The palatal process lacks a bony contact with the maxilla as in virtually all panhynobians, except *Pachyhynobius shangchengensis*, in which the pterygoid has a bony articulation with the maxilla (e.g., Clemen and Greven, 2009). The palatal process is expanded bilaterally in both the holotype and PKUP V0515, in contrast to other stem hynobiids (e.g., Dong and Wang, 1998) and *Pangerpeton* (Wang and Evans, 2006a) in which the process is narrowed distally; we thus regard an expanded palatal process as autapomorphic for *Neimengtriton*. The dorsal surface of the palatal process is grooved (Figure S2A) for receipt of the pterygoid process of the palatoquadrate as in living hynobiids (e.g., Lebedkina, 2004; Vassilieva et al., 2015). Posterior to the groove, the palatal process of the pterygoid dorsally bears a low bony ridge; based on comparisons with extant *Salamandra* (Francis, 1934), that bony ridge was the origin for the M. levator mandibulae posterior that assists in closing the jaws. The palatal process is shorter than the quadrate process in *Neimengtriton*. The otic process is even shorter, with its posterior border curved dorsally forming a trough-like groove to hold the cartilaginous ascending process of the palatoquadrate. Medially, the otic process is free from contacting any surrounding bony elements, including the parasphenoid and the otic complex (Figure S2B). The quadrate process curves posterolaterally and contributes to the jaw suspensorium by articulating with the quadrate.

Palate and braincase—The palate consists of the paired vomers, the anterior cultriform process of the parasphenoid, and the partes palatina of the paired premaxillae and maxillae. Similar to the holotype, the anteromedial fenestra in PKUP V0515 is anteriorly rounded and posteriorly constricted (Figures 3, S1, S2), opening in the anterior portion of the palate, between the vomers and the partes palatina of the premaxillae as in *Pangerpeton* and all panhynobians. As in most stem and a few living hynobiids the anteromedial fenestra of *Neimengtriton* is relatively small, with its posterior border terminating well anterior to the level of the choana (e.g., *Hynobius nebulosus*, *Pachyhynobius shangchengensis* in Fei and Ye, 2016). In other panhynobians, the fenestra is enlarged, with its posterior border either approaching (e.g., *Batrachuperus*, *Onychodactylus*, *Pseudohynobius*, *Protohynobius*, *Ranodon*, *Salamandrella* in Fei and Ye, 2016; Jiang et al., 2018) or even surpassing (e.g., *Regalerpeton* in Rong, 2018) the level of the choana.

The vomers (Figures S1D, S2B) are paired, irregular, flat bony plates that are greatly expanded laterally, each with their posterolateral border notched to form the deep, V-shaped medial margin of the choana. The vomers also are posteriorly elongate,

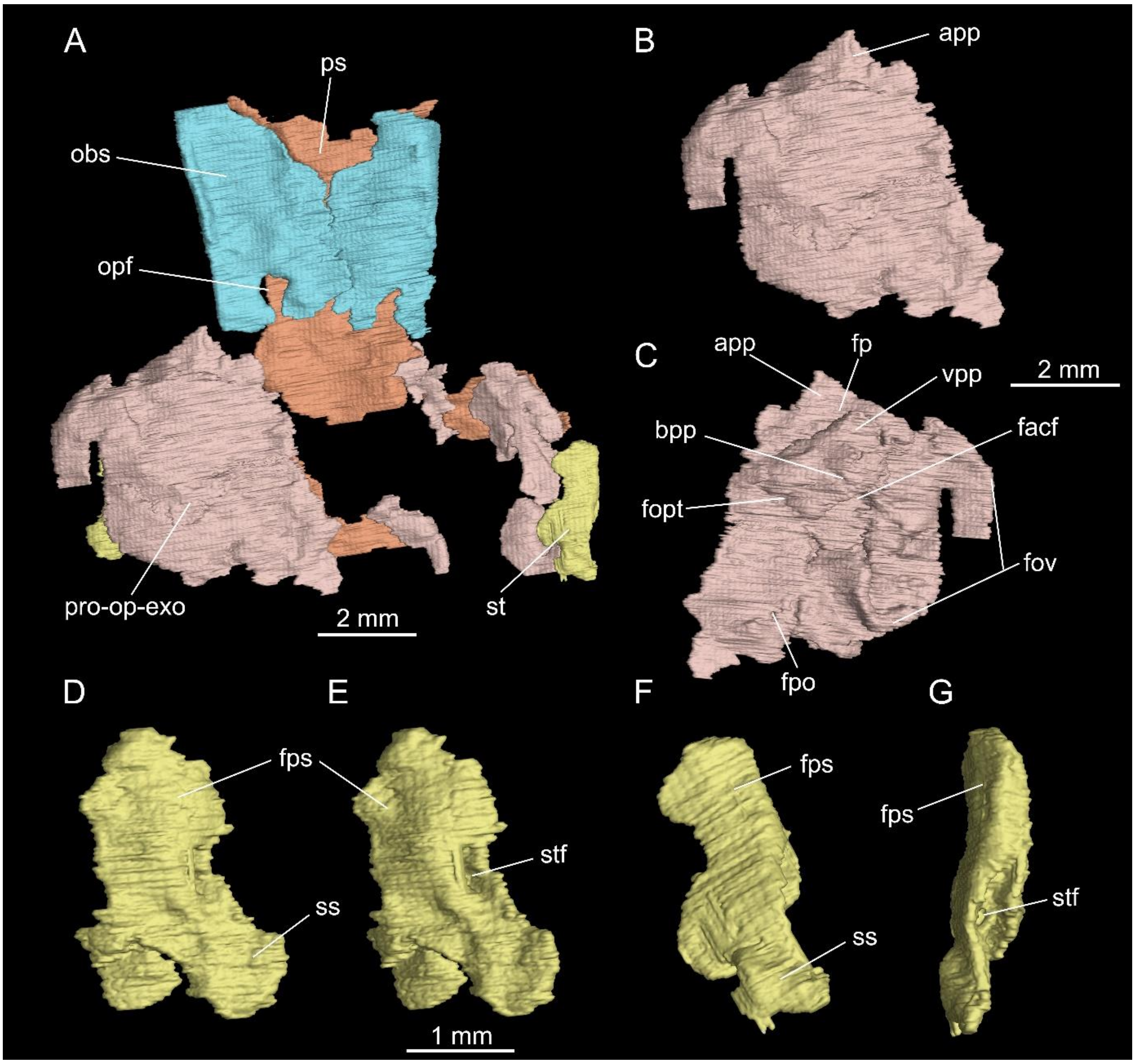

Figure S3. Micro-CT rendered reconstructions of the braincase of the newly referred specimen (PKUP V0515) of the stem hynobiid *Neimengtriton daohugouensis* comb. nov., all as preserved in slab B, Related to Figure 3. (A) braincase in dorsal view; left prootic-opisthotic-exoccipital complex in (B) dorsal and (C) ventral views; left stapes in (D) lateral and (E) anterolateral views; and right stapes in (F) lateral and (G) anterior views. See STAR Methods for abbreviations.

extending almost to the anteroposterior midpoint of the ventral surface of the skull. This differs from the condition in other panhynobians, in which the posterior end of the vomer lies relatively more anteriorly, at about one-third of the distance along the skull ventral midline (*Hynobius*, *Linglongtriton*, *Nuominerpeton*, *Onychodactylus*) or even more anteriorly (*Liaoxitriton*, *Regalerpeton*, and most other crown hynobiids; e.g., Gao et al., 2013; Rong, 2018; Fei and Ye, 2016). Therefore, we regard extreme posterior expansion of the vomer as an autopomorphy of *Neimengtriton*. The paired vomers are in elongate and straight contact with one another along the skull midline, posterior to the anteromedial fenestra. Each vomer also articulates anterolaterally with the partes palatina of the premaxilla and maxilla and posteriorly with the cultriform process of the parasphenoid. Posterior to the choana, the vomer bears a prominent and subpointed postchoanal process (retrochoanal process in Rose, 2003) extending laterally. Similar to the holotype, the vomer in PKUP V0515 ventrally bears a row of closely packed teeth close to the posterior border. Starting from the posteromedial corner of the bone, each vomerine tooth row extends anterolaterally in a shallow and anteriorly convex arc nearly to the lateral end of the postchoanal process. The vomerine tooth rows are not continuous across the skull midline. For further details on the vomerine teeth, see our Dentition account. Posterior to the vomerine tooth row, the posterior process of the vomer is extremely short, with its posterior end terminating at roughly the same level as the anterior border of the orbitosphenoid (Figure S2B).

The parasphenoid (Figures S1D, S2B) is singular, stout, and anteroposteriorly short, being only slightly longer than the vomer. The cultriform process of the parasphenoid is trapezoidal, being narrowest posteriorly and progressively broadening anteriorly, and with its anterolateral corners slightly diverging from each other bilaterally. An anteriorly broadened cultriform process also is present in *Pangerpeton* (Wang and Evans, 2006a), but in other stem (e.g., *Liaoxitriton* in Dong and Wang, 1998; *Linglongtriton* in Jia and Gao, 2019) and living panhynobians (e.g., Jiang et al., 2018) the cultriform process narrows anteriorly. Both the relatively short parasphenoid and its anteriorly broadened cultriform process are treated here as autopomorphic for *Neimengtriton*. CT images show that the lateral borders of the parasphenoid are slightly curved dorsally for articulating with the paired orbitosphenoids. The alae of the parasphenoid are mediolaterally short, extending laterally and articulating dorsally

with the otic complex. A large part of the parasphenoid between the paired alae is lost in PKUP V0515 (Figure S2B), so it remains unclear whether the parasphenoid is perforated in that region by internal carotid arteries as in *Pangerpeton* and other stem hynobiids or resembles the derived hynobiids *Pseudohynobius jinfo* (e.g., CIB 85290) and *Protohynobius puxiongensis* (e.g., CIB 98264) in having the internal carotid arteries pass through the fissure between the parasphenoid and the otic complex. The posterior process of the parasphenoid is short, articulating dorsally with the otic complex to floor the foramen magnum.

The orbitosphenoids (Figures S1D, S2B, S3A) are paired bony plates, forming the anterolateral walls of the braincase. The ventral border of the orbitosphenoid is flat and articulates ventrally with the parasphenoid, whereas the dorsal border is slightly arched dorsally and articulates with the posterior portion of the frontal and the anterior portion of the parietal. As in *Pangerpeton* and other stem hynobiids, the orbitosphenoid in *Neimengtriton* lacks the anteroventral process, which occurs in living hynobiids as a short, blunt bony process that extends medially along the anteroventral border of the orbitosphenoid (e.g., Jiang et al., 2018; Jia et al., 2019). The posterior border of the orbitosphenoid is deeply notched for the optic foramen (Figures S1D, S3A), through which passes the optic nerve (CN II) and, more posteriorly, the oculomotor nerve (CN III) would have innervated the cartilages between the orbitosphenoid and the otic complex. Both of those features occur in crown and stem hynobiids (Gao et al., 2013; Jia and Gao, 2016a; 2019; Rong, 2018) and are diagnostic for panhynobians (Jia and Gao, 2019).

On each side, the prootic fuses with the opisthotic and exoccipital into a single otic complex (Figures S1B, S1D, S2, S3). The otic complex has been dorsoventrally flattened postmortem and, as a result, both its prominentia semicircularis anterioris and prominentia semicircularis posterioris are expressed as extremely low ridges along the dorsal surface. The anterior process of the prootic is short, triangular, and directed anteromedially (Figure S3B, S3C) to articulate with the parietal. In ventral view, the ventral process of the prootic is short and triangular, extending from the anteroventral border of the prootic and overlapping the parasphenoid (Figure S3A, S3C). Despite deformation of the braincase, an open concavity between the anterior and ventral processes of the prootic is recognizable as a narrow fissure (Figure S3C), within which would open the foramen prooticum to house the Gasserian

ganglion of the trigeminal nerve (CN V) as in *Salamandra* (Francis, 1934). Posterolateral to the ventral process, the prootic ventrally bears a short and stout basal process that is directed anterolaterally (Figure S3C). The basal process is penetrated both medially by the foramen palatinum (Figure S3C) for passage of the palatine branch of the facial nerve (CN VII) and laterally by the foramen facialis (Figure S3C) for passage of the hyomandibular branch of the same nerve (CN VII). The otic complex is laterally notched for the fenestra ovalis, which is covered by the footplate of the stapes (Figure S2B). The medial wall of the prootic-opisthotic-exoccipital complex is crushed into fragmentary and flat bony plates that obscure our view of foramina transmitting auditory nerves (CN VIII). CT images show that for the left otic complex in slab B of PKUP V0515, the foramen post-oticum penetrates the base of the occipital condyle (Figure S3C) to conduct the glossopharyngeus-vagus nerves (CN IX + X) as in *Salamandra* (Francis, 1934).

The stapes (Figures S1D, S2B, S3A, S3D–3G) consists of a stylus fused with the middle part of the footplate, that latter of which covers the fenestra ovalis of the otic complex. In lateral view (Figure S3D, S3F), the footplate is oval and anteroposteriorly elongate, with its anterior, dorsal, and posterior borders articulating with the otic complex. CT images show that the footplate is convex laterally and concave medially (Figure S3D–3G) as commonly seen in salamanders. The stylus of the stapes (Figure S3A, S3F) is short and slightly constricted at the base, with a blunt distal end that would have attached to the squamosal via the ligamentum squamoso-columellare as in living salamanders (Kingsbury and Reed, 1909). Interestingly, the stylus of the stapes is perforated at the base by the stapedial foramen (Figure S3E, S3G) for passage of the stapedial artery (Goodrich, 1930) and/or hyomandibular ramus of the facial nerve (CN VII; Schmalhausen, 1968). The stapedial foramen is absent in other stem hynobiids, but occurs in several living hynobiids (e.g., *Batrachuperus londongensis*, *Ranodon*, *Salamandrella* in Jiang et al., 2018; *Hynobius amjiensis* in e.g., ZMNH AA868) and in fossil basal salamandroids (Jia and Gao, 2016b). The stapes is relatively larger in *Neimengtriton* than in other stem hynobiids (*Linglongtriton* and *Nuominerpeton* in Jia and Gao, 2016a; 2019) for which that element is known.

Mandible—The ossified mandible (Figures S1, S2) consists of the dentary, prearticular, angular, and articular. The mentomeckelian is fused with the dentary at the jaw symphysis and

is expressed as a short mental process that projects posteriorly. The coronoid is absent in the holotype and PKUP V0515, presumably because it was resorbed during development as in metamorphosed living salamanders (e.g., Lebedkina, 2004).

The dentaries (Figures S1B, S1D, S2) are paired, and elongate, forming most of the ventral and lateral aspects of the mandible. The dentary is arch-like in dorsal or ventral view and is relatively more curved along its anterior half in both the holotype and PKUP V0515, as in *Pangerpeton* (Wang and Evans, 2006a), but unlike that in other stem hynobiids (Dong and Wang, 1998; Jia and Gao, 2016a; 2019; Rong, 2018), where the paired dentaries are more constricted medially. Both dentaries in PKUP V0515 are split horizontally, with their upper and lower portions preserved on slabs A and B, respectively (Figures S1, S2). As shown by the bony impression preserved on slab B and CT images, the left and right dentaries are labially convex and their labial surfaces have a narrow, elongate dentary groove (Figure S2B) that extends anteriorly from a point opposite the anterior end of the prearticular to about one-quarter the length of the dentary from the symphysis. Anterior to the dentary groove, the labial surface of the left dentary additionally has another short anteroposteriorly oriented groove and one or two mental foramina (Figure S2B). The dentary groove and mental foramina are for passage of the external branches of the ramus mandibularis nerve (CN V_3) and its associated blood vessels, as was interpreted for the stem hynobiid *Linglongtriton* (Jia and Gao, 2019). Lingually, the dentary bears a row of closely packed teeth (see dentition account, below) that extend from the symphysis to a level corresponding to the anterior end of the angular.

The prearticular (Figures S1B, S1D, S2) is elongate, curved, and articulates along about the posterior half of the lingual surface of the dentary. The prearticular tapers anteriorly and rises dorsomedially, where its posterior part is developed as raised coronoid process bearing a dorsally concave facet for the insertion of the adductor mandibulae internus as in living hynobiids (e.g., Jiang et al., 2018; Jia et al., 2019). Below the coronoid process, the prearticular is notched along its ventral border which, in combination with the adjacent angular, forms the angular foramen (Figure S1B) to conduct the mylohyoid branch of the ramus mandibularis nerve (CN V_3 in Wilder, 1903) as in living salamanders. Posterodorsal to the angular foramen, the prearticular is penetrated by the inferior dental foramen (Figure S1B)

to transmit the inferior alveolar ramus of the facial nerve (CN VII) and the alveolar artery as in *Salamandra* (Francis, 1934).

The angular (Figures S1B, S1D, S2) is a nearly straight, elongate bony splint, about one-half the length of the prearticular, wedging between the dentary and the prearticular. Similar to the prearticular, the angular is tapered anteriorly and mediolaterally expanded posteriorly.

The articular (Figures S1B, S1D, S2) is ossified as a rectangular bone at the posterior end of the lower jaw. The articular on both sides of the skull is dislocated postmortem medially to the posterior end of the prearticular, but would have articulated laterally with the dentary, medially with the prearticular and ventrally with the angular when the individual was alive. The articular is much shorter than the angular, and is preserved lying medial to the angular on both sides of the skull in PKUP V0515. The originally (Wang, 2004a) interpreted articular in the holotype was later re-interpreted (Wang et al., 2008) as the prearticular. However, whether the holotype has an ossified articular remains unclear, because its mandible is preserved as ventral impressions. The articular is an endochondral bone that is ossified in large individuals of the stem hynobiid *Nuominerpeton* (Jia and Gao, 2016a), remains cartilaginous in the stem hynobiid *Linglongtriton* (Jia and Gao, 2019), and its condition remains unclear in the stem hynobiids *Liaoxitriton* and *Regalerpeton* and the related *Pangerpeton* (Dong and Wang, 1998; Wang and Evans, 2006a; Rong, 2018). In crown hynobiids, the articular is present as a bony element in most living taxa, but is absent in *Pseudohynobius* (e.g., CIB 17342), *Protohynobius* (e.g., CIB 98264) and *Liua tsinpaensis* (e.g., CIB 18349).

Dentition—Although not apparent in our figures, marginal teeth are present on the premaxilla, maxilla, and dentary. Teeth in the upper jaw and dentary teeth are embedded in matrix in slabs B and A, respectively, and are not observable under the microscope. CT images show that marginal teeth are tiny and closely packed in a single row along the partes dentalis of the premaxilla and maxilla and along the dental parapet of the dentary. However, the resolution of the CT images is not high enough to resolve the number of tooth positions on each element, whether the marginal teeth are pedicellate or non-pedicellate, and whether the tooth crowns are monocuspid or bicuspid.

Near their posterior edges, the paired vomers each bear an anterolaterally oriented and slightly curved row of small teeth. Transverse sections of tooth bases recognized in CT

images indicate that vomerine tooth rows contain 12–14 teeth. As for the marginal dentition, the resolution of the CT scans is not high enough to determine whether the vomerine teeth are pedicellate or non-pedicellate and have monocuspid or bicuspid crowns.

Hyobranchial apparatus

The hyobranchium of *Neimengtriton* is largely cartilaginous, with only the paired hypobranchials II and ceratobranchials II and the posteromedian basibranchial II being preserved as ossified elements in both PKUP V0515 (Figures S1D, S2B) and the holotype (Wang, 2004a: figure 2; Sullivan et al., 2014: figure 6). A similar ossification pattern occurs in the stem hynobiids *Nuominerpeton* and *Linglongtriton* (Jia and Gao, 2016a; 2019) and several living hynobiids (e.g., *Salamandrella* in Xiong et al., 2013b).

In PKUP V0515, the paired hypobranchials II and ceratobranchials II (Figures S1, S2) are subequal in length and rod-like, with their shafts weakly constricted and their anterior and posterior ends slightly expanded. Both elements lie in an oblique row, with the posterior end of the former articulated with the anterior end of the latter. In dorsal or ventral view, both are shallowly curved in opposite directions: hypobranchial II curves posteromedially and ceratobranchial II curves anterolaterally. Aside from showing less expansion of their anterior and posterior ends, these two elements appear identical in the holotype.

In PKUP V0515, basibranchial II (Figures S1D, S2B) is anteroposteriorly short, mediolaterally wide, and anchor-shaped, consisting of a pair of elongate anterolateral processes extending anterolaterally and a short median process extending anteriorly. The holotype has a similar basibranchial II, except that its anterolateral processes are relatively shorter (Wang, 2004a: figure 2). Differences in the relative lengths of the anterolateral processes in the smaller holotype versus the larger PKUP V0515 are consistent with ossification of basibranchial II increasing during development. An anchor-shaped basibranchial II was recently recognized as a plesiomorphic feature within Urodela (Jia and Gao, 2016b).

Axial skeleton

Axial skeletons in both the holotype (Wang, 2004a: figures 1, 2) and PKUP V0515 (Figures 2,

3) of *Neimengtriton* are articulated. In PKUP V0515, the axial skeleton anterior to the last caudosacral is dorsoventrally compressed, whereas the remaining vertebrae are rotated about 90° around the anteroposterior axis from their natural positions and are preserved in lateral aspect. The axial skeleton in PKUP V0515 is split through its bones, with portions of all elements preserved on slabs A and B. The axial skeleton in PKUP V0515 consists of an atlas, 15 trunk, one sacral, four caudosacral, and 29 caudal vertebrae. By contrast, the holotype has suffered less rotation of its tail, and its vertebrae and ribs are largely represented by ventral impressions. Like PKUP V0515, the holotype has an atlas, 15 trunk vertebrae, and a sacral vertebra (Wang 2004a). Because he did not differentiate between caudosacrals and caudals, Wang (2004a) reported 32 preserved caudals in the holotype. Recently those postsacral vertebrae were reinterpreted as seven caudosacrals and 25 caudals (Jia and Gao, 2019; see below). The exact number of caudals in the holotype is unknown, because the end of its tail is missing.

The atlas in PKUP V0515 (Figures 2, 3, S1, S2) is hourglass shaped in dorsal or ventral outline, and it is slightly broader than the trunk vertebrae. The odontoid process of the atlas is subtriangular in ventral outline, projecting anteriorly for a short distance from the lower portion of the anterior midline of the centrum (Figure S1B). The paired anterior cotyles lie dorsolateral to the odontoid process. The anterior cotyles are shallowly compressed dorsoventrally and oval in anterior outline, with shallowly concave articular surfaces. The posterior cotyle is subcircular in posterior outline and deeply concave. CT images show the atlantal centrum is indented by a ventromedian depression that is bordered on either side by a shallow bony ridge extending anterolaterally along the underside of the centrum. On each side, at the junction between the anterior portion of the neural arch wall and the centrum, the atlas is penetrated by a foramen for passage of the first spinal nerve as in all urodeles (e.g., Edwards, 1976; Jiang et al., 2018; Jia and Gao, 2019). The neural arch is tall and, along its dorsomedian surface, its neural spine extends anteroposteriorly as a shallow bony ridge. The atlas lacks transverse processes or ribs as in other panhynobians (e.g., Xiong et al., 2013a; Jia et al., 2019).

All postatlantal vertebrae have amphicoelous centra. No spinal nerve foramina penetrate the neural arch, indicating that spinal nerves pass through the fissure between successive

vertebrae as in living cryptobranchoids (e.g., Edwards, 1976; Naylor, 1978; Zhang, 1985; Xiong et al., 2013a). The anterior three trunk vertebrae are slightly shorter than the remaining twelve trunk vertebrae, the latter of which are similar in size to one another (Figures 2, 3). The trunk vertebrae are preserved tightly articulated with one another, with their prezygapophyses and postzygapophyses having similar sized and oval-shaped facets. The centrum is smooth ventrally, lacking subcentral foramina or subcentral keels as in *Pangerpeton* (PKUP V0218) and other panhynobians (e.g., Xiong et al., 2013a; Jiang et al., 2018). The neural arch is high and dorsally bears a shallow neural spine. Trunk vertebrae bear transverse processes that are single-headed (unicapitate), straight, short, and directed posterolaterally to articulate with their corresponding ribs.

Ribs in the trunk region (Figures 2, 3) are unicapitate, with their proximal head expanded for articulation with the complementary transverse processes on the vertebrae. Most ribs are straight, although several are curved along the middle part of the trunk region. Compared to more posterior ribs, the first three pairs are more expanded to serve as attachments for the M. thoracic-scapularis as in *Salamandra* (Francis, 1934). The first nine pairs of ribs are similar in length, but the posterior six gradually decrease in length, with the last pair being little more than triangular bony struts.

The sacral vertebra (Figures 2, 3) is subequal in length to the last trunk vertebra, but differs in bearing stouter transverse processes. The pair of sacral ribs are strongly built and similar in length to the first nine pairs of trunk ribs.

Following the criteria of Wake and Dresner (1967), who recognized the last caudosacral vertebra in salamanders as the first postsacral bearing a haemal arch, we count four caudosacrals in PKUP V0515 (Figures 2, 3), compared to seven caudosacrals in the holotype. As in living and other stem hynobiids, the first caudosacral is the largest and the last caudosacral is the smallest (e.g., Jiang et al., 2018; Jia and Gao, 2019). The haemal spine on the last caudosacral vertebra is elongate, straight, and extends posteroventrally to form an angle with its centrum that is smaller than in any of the caudal vertebrae. As shown in slab B of PKUP V0515, the first three caudosacrals bear free ribs (Figures 2, 3) that are slender, straight, and similar in length with one another.

Caudal vertebrae (Figures 2, 3) gradually decrease in size posteriorly. Most caudal

vertebrae have well-developed neural and haemal arches, however, the last seven caudals in PKUP V0515 are preserved as irregular bony elements. In the last four caudal vertebrae, the ossified part of each is a tiny bony knob that likely represents the centrum, because the bony knob lies more ventrally within the tail compared to the expected position of the corresponding neural arch. If our interpretation is correct, this indicates that centra in *Neimengtriton* ossified earlier than their corresponding neural arches. Such an ossification sequence is a derived feature of urodeles that has been documented in the living hynobiid *Salamandrella* and salamandroids (Boisvert, 2009). However, the reverse sequence, in which the centrum ossifies later than the neural arch, occurs as a plesiomorphic developmental pattern in temnospondyls and the hynobiid "*Ranodon tsinpaensis*" (Boisvert, 2009; but see Dando et al., 2019 for alternative interpretations regarding the ossification pattern for "*Ranodon tsinpaensis*" reported by Boisvert, 2009).

Appendicular skeleton

Pectoral girdle and forelimb—The pectoral girdle and forelimbs are complete and largely articulated in PKUP V0515, and both are more robust than in the holotype. The scapula and coracoid (Figures 2, 3) are fused into the scapulocoracoid as commonly seen in non-sirenid salamanders (e.g., Duellman and Trueb, 1986). The blade-like scapula is trapezoidal in outline, with its dorsal border wider than its ventral border. The scapula is shortened dorsoventrally, being slightly over one-half the width of the coracoid plate in both the holotype and PKUP V0515. By contrast, the scapular height is similar to or slightly longer than the width of the coracoid plate in other stem hynobiids, other hynobiid-like taxa (*Laccotriton* and *Sinerpeton*), and basal salamandroids (*Beiyanerpeton* and *Qinglongtriton*; Zhang et al., 2009; Gao and Shubin, 2012; Jia and Gao, 2016a; 2016b; 2019). We thus regard the shortened scapula as autopomorphic for *Neimengtriton*. The dorsal border of the scapular blade is straight, indicating that the suprascapular remains cartilaginous as in *Pangerpeton* and other stem hynobiids (e.g., Wang and Evans, 2006a; Rong, 2018). The coracoid plate is ventrally expanded, having a straight anterior border and convex medial and posterior borders. The procoracoid remains cartilaginous. The paired scapulocoracoids in PKUP V0515 are broken transversely at the level of the glenoid fossa; thus, both the size of the glenoid

fossa and the position of the supracoracoid foramen are unrecognizable. Judging by published images of the holotype (Wang, 2004a: figure 2; Sullivan et al., 2014: figure 6), the supracoracoid foramen lies in the middle of the coracoid plate and directly below the scapular blade, whereas the glenoid fossa is posteroventral to the scapular blade.

The humerus is straight and is almost twice the length of the ulna and radius (Figures 2, 3). The humeral shaft is moderately short and robust. Both the proximal and distal ends of the bone are expanded, with the latter being larger than the former. As shown in the right forelimb of PKUP V0515 (Figure 3), the extensor side of the proximal end of the humerus bears a crista dorsalis developed as a small, subtriangular bony knob, onto which inserts the M. subscapularis as in *Salamandra* (Francis, 1934). On the flexor side of the humerus, the crista ventralis is a large and subtriangular bony crest for attachment of the M. pectoralis and M. supracoracoideus as in *Salamandra* (Francis, 1934). Distally, the humerus bears a radial condyle and an ulna condyle, both subequal in size and which articulate with the radius and ulna, respectively.

Similar to the humerus, both the radius and ulna have a short shaft and expanded proximal and distal ends (Figures 2, 3). The radius is slightly shorter than the ulna. The radius is more expanded distally than proximally, whereas the ulna is similar in size at both ends. The distal end of the radius bears an oblique facet for articulation with the ossified intermedium and centrale 1 (proximal centrale of Holmgren, 1933) and, possibly, with the unossified radiale as in living hynobiids (e.g., Jia et al., 2019). The olecranon process of the ulna is not ossified. The ulna articulates distally with the ulnare and the intermedium, but does not contact centrale 1.

Both forelimbs in PKUP V0515 have seven ossified carpals, with the unpreserved preaxial components (element y and radiale or a fusion of both) presumably remaining cartilaginous (Figures 2, 3; S4A). For the proximal carpals, the intermedium in the left forelimb is dislocated towards the nearby ulna, but that element remains in situ in the right forelimb, where it is wedged between the radius and the ulna. The ulnare is the largest bony carpal element; it articulates proximally with the ulna and distally with distal carpal 4. There are two centralia in each side of the manus. The two centralia are arranged along the proximodistal axis of the manus, with centrale 1 articulating proximally with the intermedium

and centrale 2 articulating distally with the basale commune and distal carpal 3. Within the digital arch, the basale commune articulates distally both with the metacarpals I and II, as a common pattern in urodeles (Shubin and Wake, 2003). Distal carpals 3 and 4 are subequal in size and articulate distally with metacarpals III and IV, respectively. The carpals are less ossified in the holotype (Wang, 2004a: figure 2): two bony elements are preserved associated with metacarpals I–III in both the left and right manus, indicating that those two elements are affiliated with the digital arch. The anterior element is larger than the posterior element and likely represents the ossification center of basale commune, whereas the more posterior element likely represents the ossification center of distal carpal 3.

Both the left and right manus each have four digits in PKUP V0515 (Figures 2, 3, S4A) and in the intact left manus in the holotype (Wang, 2004a: figure 2). In each digit, the metacarpal and all but the terminal phalanx are similar in having a constricted and short shaft and expanded proximal and distal ends. The terminal phalanxes differ in being subtriangular, with their distal end tapered to a sharp point. The bones in each digit progressively decrease in size from the metacarpal to last phalanx. The proximal end of metacarpal I is convex, whereas the distal end of that metacarpal and the proximal and distal borders in the other three metacarpals are flat. Metacarpal III is the most robust and the longest, whereas metacarpal I is the shortest, yet still relatively robust given its size. Metacarpals II and IV are subequal in length, with the latter being the least robust of the four metacarpals. Both metacarpals II and III are not expanded anteroposteriorly either in the holotype or PKUP V0515, a common feature in living hynobiids (e.g., Jia and Gao, 2016a; Jiang et al., 2018). However, metacarpal II is commonly expanded anteroposteriorly in most stem hynobiids except *Linglongtriton*, in which metacarpal III instead is expanded anteroposteriorly (Jia and Gao, 2019). The phalangeal formula for PKUP V0515 is 2-2-4-2 in the left manus and 2-2-4-3 in the right; those contrast with the reported phalangeal formula (for the intact left manus) in the holotype of 2-2-3-2 (Wang, 2004a). In PKUP V0515, digit III is the longest and digit I the shortest in both the left and right manus. Digit II is similar in length to digit IV in the left manus, but is slightly shorter than the latter in the right manus.

Pelvic girdle and hind limb—In PKUP V0515, the pelvic girdle and both hind limbs are moderately well preserved and largely in articulation. The bony components of the pelvic

girdle (Figures 2, 3, S4B, S4C) consist of paired ilia and ischia. The prepubis and the ypsiloid presumably remained cartilaginous as in *Pangerpeton* and other stem hynobiids (e.g., Wang and Evans, 2006a; Rong, 2018). The left ilium is preserved intact and exposed on slab A, whereas the right ilium is broken and is embedded under the corresponding ischium. The ilium is club-like, with an expanded acetabular region and a narrow shaft that expands moderately towards its dorsal end. The paired ischia are partially embedded in matrix on slab B under the axial skeleton, but CT images (Figure S4B, S4C) reveal them to be large bony plates that broadly contact each other along a straight median suture. The anterior border of the ischium is convex, whereas the lateral border is concave midway along its length. The posterolateral corner of the ischium bears a stout ischial spine directed posterolaterally.

The femur (Figures 2, 3, S4D) is straight and slightly longer than the humerus. The femoral shaft is short and constricted, whereas the proximal and distal ends of the bone are expanded, with the latter more expanded than the former. The trochanter of the femur is unclear in the holotype, because both femora are preserved as natural molds (Wang, 2004a). Although embedded in matrix in slab B, CT images of the right femur in PKUP V0515 reveal the ventral surface bears a twig-like trochanter.

The tibia and the fibula (Figures 2, 3) are moderately elongate, with the former slightly

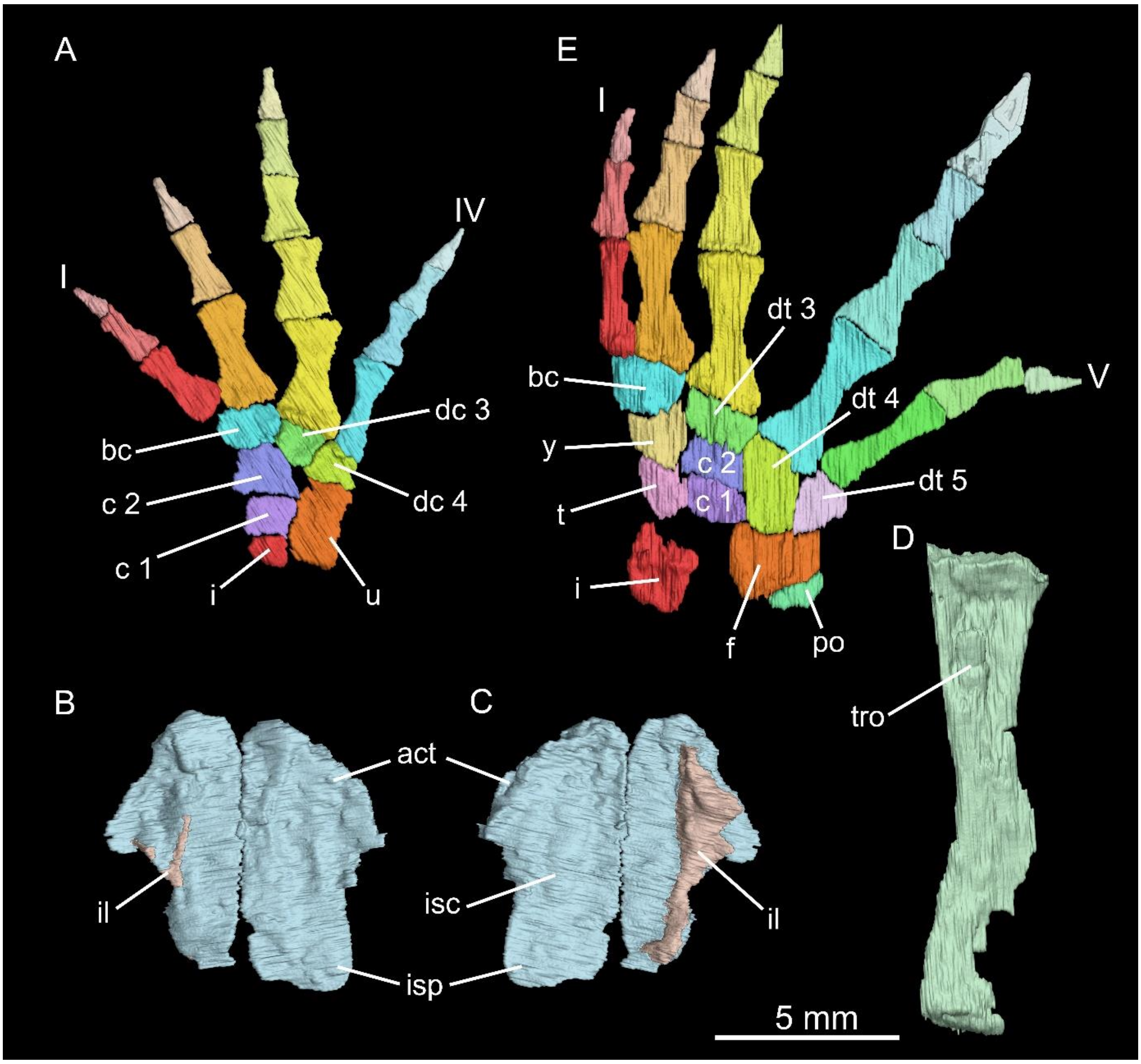

Figure S4. Micro-CT rendered reconstructions of portions of the appendicular skeleton of the newly referred specimen (PKUP V0515) of the stem hynobiid *Neimengtriton daohugouensis* comb. nov., all as preserved in slab B, Related to Figures 2, 3. Autopodium of the (A) right manus and (E) right pes, both in dorsal view; ossified pelvic girdle in (B) ventral and (C) dorsal views; and right femur in (D) ventral view. See STAR Methods for abbreviations.

---

shorter than the latter. The tibia is straight and is more expanded proximally than distally; whereas the fibula is slightly curved and more expanded distally than proximally. The tibia articulates distally with the tibiale and intermedium, but not centrale 1, whereas the fibula articulates distally with the fibulare and intermedium.

Tarsals are fully ossified in PKUP V0515, with 11 bony elements identified in each pes (Figures 2, 3, S4E). As best shown in the left pes (Figures 2B, 3B), the intermedium wedges

between the tibia and the fibular and is in contact with the tibiale preaxially, the fibulare postaxially, and the centrale 1 distally. The pattern is nearly identical in the figured right pes, except for the intermedium being slightly displaced postmortem (Figures 2B, 3B, S4E). The fibulare is the largest mesopodial element in the pes; besides its above-mentioned contacts, the fibulare articulates distally with distal tarsals 4 and 5, and variably (right pes) articulates postaxially with the postminimus. The tibiale articulates proximally with the tibial, distally with element y, and postaxially with the intermedium. Similar to the manus, the pes contains two centralia arranged along the proximodistal axis of the central column. Most stem hynobiids are known to have two ossified centralia in their pes (e.g., Dong and Wang, 1998; Jia and Gao, 2016a; Rong, 2018), except the Late Jurassic *Linglongtriton*, whose pes has a single large, bony centrale derived from fusion of two centralia (Jia and Gao, 2019). In the distal digital arch, the basale commune articulates distally with metatarsals I and II, whereas the distal tarsals 3, 4, and 5 articulate with their corresponding metatarsals III, IV, and V, respectively. Distal tarsal 4 is substantially larger than distal tarsal 5, with the former extending proximally into the central region of the pes. Interestingly, both hindlimbs have an ossified postminimus that articulates either with distal tarsal 5 (left pes) or the fibulare (right pes; Figure 3B, S4E). An ossified postminimus is plesiomorphically present in temnospondyls (Shubin and Wake, 2003) and several living hynobiid taxa (e.g., *Batrachuperus*; Jiang et al., 2018), but absent in derived salamandroids (Shubin and Wake, 2003); its presence in PKUP V0515 indicates that a bony postminimus also presents in stem hynobiids. The holotype is completely missing its right pes and its left pes preserves three ossified tarsals in a triangular arrangement (Wang, 2004a: figure 2). We provisionally identify those three tarsals as follows: the largest element lying near metatarsals I and II is the ossification center of the basale commune; the medium-sized element beside the inferred basale commune and articulating with metatarsal III likely is distal tarsal 3; and the smallest element lying more proximally and articulating with both the inferred basale commune and distal tarsal 3 is centrale 2.

Both pes in PKUP V0515 and the preserved left pes in the holotype have five digits. The metatarsals and all but the terminal phalanx have a constricted mid shaft and expanded proximal and distal ends, whereas the terminal phalanxes are subtriangular and taper distally (Figures 2, 3, S4E). Metatarsals II–IV are similar in length and robustness with one another,

and each of them is slightly longer and more robust than metatarsals I and V. Metatarsal V is slightly longer than metatarsal I. In each digit, from the metatarsal to the penultimate phalanx, the proximal border of each bony element is slightly wider than its distal border. The phalangeal formula is 2-3-3-4-2 in both the left and right pes in PKUP V0515, but is 2-2-3-4-2 in the preserved left pes in the holotype (Wang 2004a). In both the holotype and PKUP V0515, digit IV is the longest and digits III, II, V, and I are successively shorter.

Table S1 Information on Mesozoic and extant salamanders for comparative studies, Related to STAR Methods

Genus	Species	Catalogue number	Fossil or extant
		**Cryptobranchoidea**	
Andrias	*davidianus*	CNU-Z735, Z736	Extant
Cryptobranchus	*alleganiensis*	FMNH 84132, 84133	Extant
Hynobius	*amjiensis*	ZMNH AA868, AA871	Extant
Linglongtriton	*daxishanensis*	PKUP V0277	Fossil
Liaoxitriton	*zhongjiani*	PKUP V0300, V0301	Fossil
Liua	*shihi*	CIB 19910403, 19910404	Extant
Onychodactylus	*japonicus*	FMNH 195610, 285321	Extant
Pachyhynobius	*shangchengensis*	CIB 72887, 72888	Extant
Pangerpeton	*sinensis*	PKUP V0218, V0219, V0222, V0224	Fossil
Protohynobius	*puxiongensis*	CIB 97503, 97504	Extant
Paradactylodon	*mustersi*	FMNH 211936	Extant
Pseudohynobius	*flavomaculatus*	CIB 17342, 17343	Extant
Ranodon	*sibiricus*	FMNH 83050, 83051	Extant
Salamandrella	*keyserlingii*	FMNH 83525, 83526	Extant
		**Salamandroidea**	
Ambystoma	*maculatum*	FLMNH 26607 (MorphoSource)	Extant
Amphiuma	*means*	FMNH 84123, 84124	Extant
Beiyanerpeton	*jianpingensis*	PKUP V0605, V0608	Fossil
Dicamptodon	*tenebrosus*	FMNH 59264, 201005	Extant
Qinglongtriton	*gangouensis*	PKUP V0226, V0228	Fossil

See STAR Methods for institutional abbreviations.

2 Characters used for cladistic analysis, Related to STAR Methods

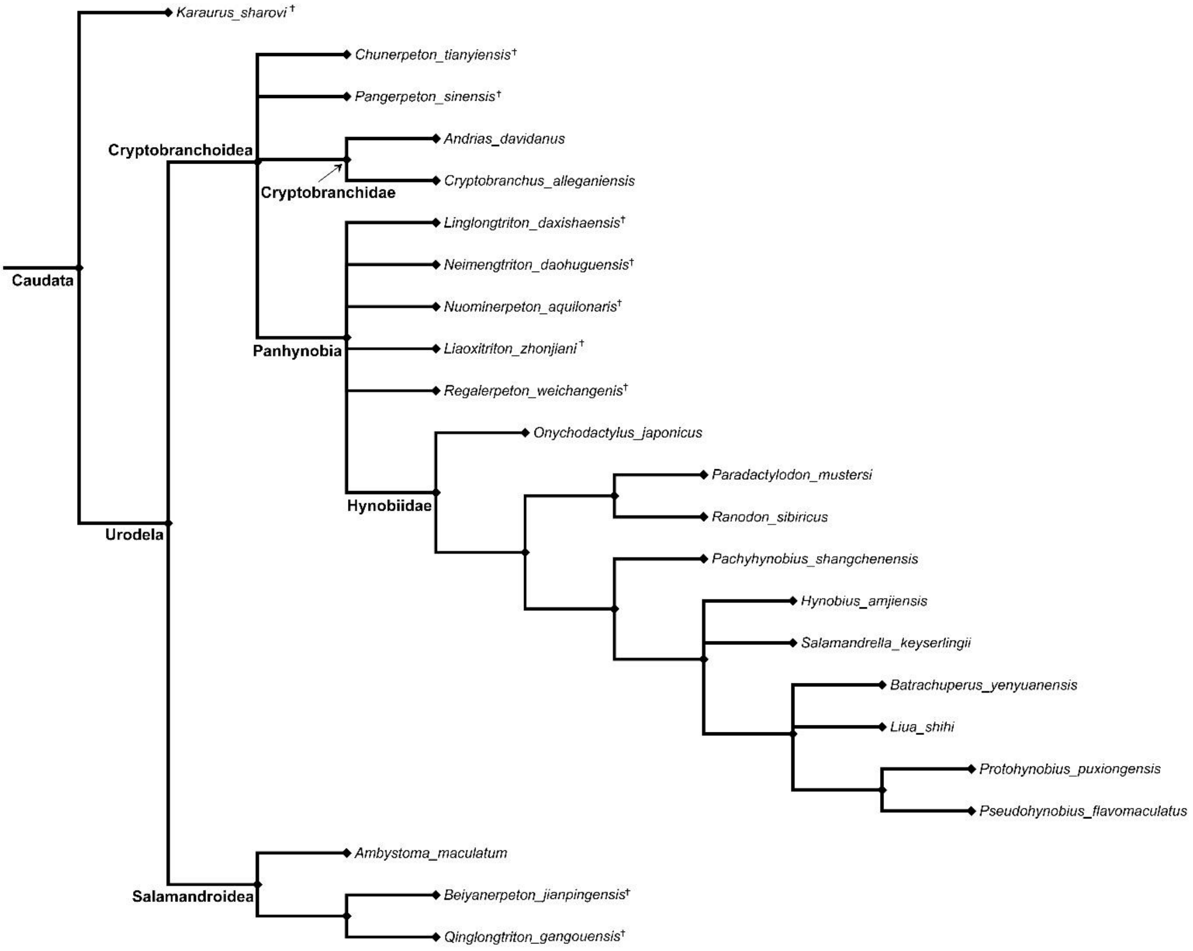

Figure S5. Consensus tree of 14 most parsimonious trees showing the interrelationships of Cryptobranchoidea with the addition of the modern salamandroid *Ambystoma maculatum*, Related to Figure 6. Note that terminal and internal branches are in the same length and fossil taxa are denoted with the cross symbol.

Characters used in the phylogenetic analyses are classified in 10 categories, including skull roof, suspensorium, palate, braincase, mandible, dentition, hyobranchium, axial skeleton, appendicular skeleton, soft tissue and developmental pattern. About 76 % (82) of the 108 characters are based on or modified from four studies (Zhao and Hu, 1984; Duellman and Trueb, 1986; Gao and Shubin, 2012; Jia and Gao, 2019), and 24 % (26) are identified as characters (denoted by asterisks) recognized in this study based on our accumulating

knowledge of and our at-hand micro-CT scan data of both extant and fossil specimens of cryptobranchoids, including 15, 18, 26, 28, 29, 32, 33, 35, 40, 48, 49, 50, 69, 70, 71, 73, 77, 81, 82, 86, 87, 90, 95, 96, 102, 108. As mentioned in previous studies (e.g., Wiens et al., 2005) and the STAR Methods, neoteny has an enormous impact on phylogeny of salamanders because taxonomically informative signals in neotenic taxa tend to be outnumbered and overshadowed by the many characters caused by neoteny. To mitigate these impacts, we score the character states as question marks for 11 characters caused by neoteny, including 4, 33, 34, 37, 38, 44, 45, 49, 50, 63, 64.

Skull roof

1. Alary process of premaxilla: (0) overlaps nasal; or (1) overlaps frontal. From Trueb, 1993 (character 16) and Jia and Gao, 2019 (character 2).
2. Anterodorsal fenestra: (0) present; or (1) absent. From Zhao and Hu, 1984 (character 4) and Jia and Gao, 2019 (character 6).
3. Anterodorsal fenestra's involvement with adjacent bones: anterodorsal fenestra is (0) bordered by the premaxilla and nasal; (1) by the premaxilla, nasal and frontal; (2) by the premaxilla and frontal; (3) enclosed within the fused/separated partes dorsalis of the premaxillae. From Jia and Gao, 2019 (character 107).
4. Maxilla: (0) present in adults as a normal element of maxillary arcade; (1) reduced to similar length as premaxilla or entirely lost and functionally replaced by a modified vomer. From Duellman and Trueb, 1986 (character C) and Jia and Gao, 2019 (character 3). [**neoteny influenced character**].
5. Anterior process of the maxilla: (0) elongated and extensively overlap the posterolateral labial surface of premaxilla; (1) rudimentary or absent, and has a blunt anterior facet abutting joint with the premaxilla. From Jia and Gao, 2019 (character 106).
6. Maxillary tooth row: (0) extending close to the posterior extremity of maxilla; or (1) terminating far anterior to the posterior extremity. From Jia and Gao, 2019 (character 4).
7. Maxilla/nasal contact: (0) absent; or (1) present. Modified from Zhao and Hu, 1984 (character 5).
8. Maxilla/frontal contact: (0) absent; or (1) present. From Gao and Shubin, 2001 (character

33) and Jia and Gao, 2019 (character 62).

9. Maxilla/pterygoid bony contact: (0) absent; or (1) present. From Jia and Gao, 2019 (character 18).
10. Septomaxilla (Duellman and Trueb, 1986: character D): (0) present; (1) absent. From Jia and Gao, 2019 (character 5).
11. Lacrimal: (0) present; or (1) absent. From Duellman and Trueb, 1986 (character F) and Jia and Gao, 2019 (character 10).
12. Lacrimal's involvement with naris and orbit: lacrimal (0) enters naris only; (1) enters orbit only; (2) enters neither naris nor orbit; (3) enters both naris and orbit. From Zhao and Hu, 1984 (character 5) and Jia and Gao, 2019 (character 108).
13. Nasolacrimal duct: (0) present; or (1) absent. From Jia and Gao, 2019 (character 57).
14. Nasal ossification (modified from Duellman and Trueb, 1986: character E): (0) present with midline contact; (1) widely separate without midline contact; (2) separate from each other by a narrow median fissure; (3) nasal absent. Modified from Jia and Gao, 2019 (character 8).
15. *Nasal anterior border: (0) smooth; (1) bifurcated to receive the alary process of premaxilla.
16. Lateral expansion of nasals: (0) same width or slightly wider than frontals; or (1) nasals greatly reduced and narrower than frontals. From Jia and Gao, 2019 (character 9).
17. Nasal/prefrontal contact: (0) absent; or (1) present. From Jia and Gao, 2019 (character 23).
18. *Nasal dimension: (0) long than wide; (1) wide than long.
19. Lateral wall of nasal capsule: (0) complete; (1) incomplete. From Trueb, 1993 (character 4) and Jia and Gao, 2019 (character 54).
20. Lateral narial fenestra: (0) absent; (1) present. From Trueb, 1993 (character 5) and Jia and Gao, 2019 (character 55).
21. Frontal anterior extension: (0) frontal does not extend lateral to nasal bone; (1) frontal does extend to lateral border of nasal. From Jia and Gao, 2019 (character 59).
22. Anterolateral process of parietal: (0) poorly defined or absent; (1) well-developed process extending to or surpassing midlevel of orbit. From Jia and Gao, 2019 (character 60).

23. Parietal/prefrontal contact: (0) absent; or (1) present. From Jia and Gao, 2019 (character 61).
24. Position of the trochlear foramen: (0) penetrates the parietal; (1) pass through the fissure between parietal and orbitosphenoid; (2) penetrates the orbitosphenoid. From Jia and Gao, 2019 (character 109).
25. Frontoparietal fontanelle: (0) absent (1) present. From Trueb and Cloutier, 1991 (char. 16) and Jia and Gao, 2019 (character 119).
26. *Frontoparietal fontanelle size: (0) large; (1) small.

Suspensorium

27. Medial contact of squamosal with parietal or other roofing element: (0) contact present; (1) contact absent. From Gao and Shubin, 2001 (character 37) and Jia and Gao, 2019 (character 66).
28. *Quadrate: (0) distally slender; or (1) distally greatly expanded.
29. *Nerve foramina on quadrate: (0) present; (1) absent.
30. Sculptured dermal skull roof: (0) present as heavily sculptured surface covering; (1) weakly sculptured or absent. From Jia and Gao, 2019 (character 58).

Palate

31. Anteromedial fenestra: (0) present; (1) absent. From Jia and Gao, 2019 (character 7).
32. *Anteromedial fenestra size: (0) less than or equal to one third length of vomer; (1) more than one third the length of vomer.
33. *Vomer: (0) contact medially; (1) widely separated medially from each other; or (2) narrowly separated from each other by a narrow median suture. [**neoteny influenced character**]
34. Posterior process of vomer: (0) present as an elongated process overlapping or extending along the parasphenoid; (1) shortened as a rudimentary process contacting the anterior part of the parasphenoid; or (2) no prominent posterior process. From Jia and Gao, 2019 (character 13). [**neoteny influenced character**]
35. *Post-choanal process of vomer: (0) poorly developed; (1) well-developed as a prominent

process tapering laterally.

36. Posterolateral border of vomer: (0) not notched; (1) slightly concave for choana; (2) deeply notched. From Jia and Gao, 2019 (character 14).
37. vomerine teeth location: (0) vomerine teeth confined to the anterolateral margin of the vomer; or (1) located at the middle portion of the vomer; or (2) located at the posterior portion of the vomer. Modified from Zhao and Hu, 1984 (character 2) [**neoteny influenced character**]
38. Vomerine tooth row direction: (0) present as a transverse or oblique tooth row; (1) parallel with the maxillary arcade; (2) single, elongated vomerine tooth row longitudinally arranged along the posterior process of the vomer. Modified from Duellman and Trueb, 1986 (character S). [**neoteny influenced character**]
39. For transversely arranged vomerine teeth: (0) inner branch longer than outer branch; or (1) similar in length; (2) outer branch longer than inner branch. Modified from Zhao and Hu, 1984 (character 3).
40. *Length of the cultriform process of the parasphenoid: (0) much longer than vomer; (1) shortened close to the length of vomer.
41. *Cultriform process of the parasphenoid: (0) wider anteriorly than posteriorly; (1) narrower anteriorly than posteriorly or being roughly the same in width.
42. Internal carotid foramen penetrating parasphenoid: (0) present; or (1) absent. From Jia and Gao, 2019 (character 21).
43. Parasphenoid/pterygoid contact: (0) contact at the base of parasphenoid; (1) contact along the anterior extension of parasphenoid; (2) contact absent. From Jia and Gao, 2019 (character 44).
44. Palatine in adult: (0) present as discrete element; (1) absent by loss or fusion in adult. From Jia and Gao, 2019 (character 19). [**neoteny influenced character**]
45. Multiple and parallel rows of palatine teeth in adult: (0) absent; (1) present. From Jia and Gao, 2019 (character 38). [**neoteny influenced character**]
46. Shape of pterygoid: (0) triradiate and boomerang-shaped; (1) enlarged with distinct anteromedial process suturing with parasphenoid. From Jia and Gao, 2019 (character 16).
47. pterygoid teeth: (0) present; (1) absent. From Jia and Gao, 2019 (character 39). [**neoteny**

influenced character]

48. *Anterolateral process of pterygoid: (0) enlarged bilaterally; or (1) tapering as a slender bar.
49. *Length of anterolateral process of pterygoid: (0) shorter or similar to; or (1) longer than the length of the posterior ramus of pterygoid. [**neoteny influenced character**]
50. *Direction of anterolateral process of pterygoid: (0) anterolaterally; (1) anteromedially. [**neoteny influenced character**]
51. Ossification of the ascending process of the palatoquadrate: (0) absent; (1) present. From Jia and Gao, 2019 (character 110).

Braincase

52. Orbitosphenoid ossification: (0) present; (1) absent. From Jia and Gao, 2019 (character 43).
53. Anteroventral extension of the orbitosphenoid on the ventral border: (0) present; (1) absent. From Jia and Gao, 2019 (character 111).
54. Position of the optic foramen: (0) opens at the posterior border of the orbitosphenoid; (1) opens within the orbitosphenoid; (2) penetrates the cartilaginous plate posterior to the orbitosphenoid. From Jia and Gao, 2019 (character 112).
55. Position of the oculomotor foramen: (0) opens at the posterior border of the orbitosphenoid; (1) opens within the orbitosphenoid; (2) penetrates the cartilaginous plate posterior to the orbitosphenoid. From Jia and Gao, 2019 (character 113).
56. Prootic/exoccipital/opisthotic complex: (0) three elements separate; (1) exoccipital/opisthotic fused with free prootic; (2) all three elements fused. From Jia and Gao, 2019 (character 12).
57. Midline contact of otic-occipital complex over foramen magnum: (0) absent; or (1) present. From Jia and Gao, 2019 (character 65).
58. Operculum: (0) absent; or (1) present. From Jia and Gao, 2019 (character 53).
59. Ossified stapes: (0) present in adults; (1) absent in adults. From Duellman and Trueb, 1986 (character K) and Jia and Gao, 2019 (character 52).
60. Stapedial foramen at the base of the stylus of the stapes: (0) present; (1) absent. From Jia

and Gao, 2019 (character 114).

Mandible

61. Dentary groove on the labial aspect of the dentary: (0) present; (1) absent. From Jia and Gao, 2019 (character 115).
62. Angular/prearticular fusion: (0) angular present as a separate element; (1) angular fused to prearticular. From Duellman and Trueb, 1986 (character Q) and Jia and Gao, 2019 (character 26).
63. Coronoid (modified from Trueb, 1993: character 22): (0) present in adult stage as a separate element; (1) absent in adult stage. From Jia and Gao, 2019 (character 27). [**neoteny influenced character**]
64. Coronoid teeth in adult: (0) absent; (1) present. From Jia and Gao, 2019 (character 28). [**neoteny influenced character**]
65. Articular: (0) present as separate bony element; (1) absent by fusion with neighboring bones or remain cartilaginous. From Jia and Gao, 2019 (character 29).

Dentition

66. Marginal teeth (Parsons and Williams, 1962): (0) nonpedicellate; (1) pedicellate. From Jia and Gao, 2019 (character 41).
67. Tooth crown: (0) monocuspid; (1) bicuspid. From Jia and Gao, 2019 (character 42).

Hyobranchium

68. Radial loop: (0) absent; or (1) present. From Zhao and Hu, 1984 (character 9).
69. *Paired radial loops: (0) crossed as figure eight; or (1) crossed as “O”-shaped.
70. *Radial loop/ceratohyal contact: (0) fused with each other; or (1) articulates but not fused with each other.
71. *Cornua: (0) absent; (1) present as plate; or (2) annular-like element.
72. Ceratohyal ossification: (0) absent; (1) present. From Jia and Gao, 2019 (character 117).
73. *Hypohyal: (0) absent; or (1) present.
74. Hypobranchial I and ceratobranchial I: (0) two elements remain separate; (1) two

elements fused. From Jia and Gao, 2019 (character 32).

75. Ossification of hypobranchial I: (0) present; or (1) absent. From Jia and Gao, 2019 (character 33).
76. Ceratobranchial II ossification in adults: (0) present; or (1) absent. From Jia and Gao, 2019 (character 34).
77. *Ceratobranchial III and IV: (0) present either as ossified or cartilaginous; or (1) absent (1).
78. Basibranchial II: (0) present as ossified or cartilaginous; (1) absent. From Jia and Gao, 2019 (character 35).
79. Basibranchial II shape: (0) anchor shaped; (1) non-anchor shaped. From Jia and Gao, 2019 (character 116).

Axial skeleton

80. Tuberculum interglenoideum of atlas: (0) absent; (1) present. From Jia and Gao, 2019 (character 47).
81. *Transverse process of atlas: (0) absent; (1) present.
82. *Caudosacral vertebrae number: (0) 5 or more; (1) 4; (2) 3 or less.
83. Atlantal spinal nerve foramen: (0) absent; (1) present. From Jia and Gao, 2019 (character 50).
84. Head of postatlantal ribs: (0) bicapitate; or (1) unicapitate. From Jia and Gao, 2019 (character 48).
85. Postatlantal spinal nerve foramina: (0) all postatlantal spinal nerves exit intervertebrally; (1) spinal nerve foramina present on posterior caudal vertebrae; (2) foramina present on all caudal vertebrae; (3) foramina occur in trunk, sacral and caudal series. From Hecht and Edwards, 1977 and Jia and Gao, 2019 (character 51).

Appendicular skeleton

86. *Ratio between the length of scapular and coracoid along the proximodistal axis: (0) <= 1; or (1) > 1.
87. *Coracoid plate anteroposterior extension: (0) not obvious; (1) obvious.

88. Dorsal and ventral crests of humerus: (0) poorly defined; or (1) well developed. From Jia and Gao, 2019 (character 69).
89. Femoral trochanter forming a twig-like projection: (0) absent; (1) well developed as a twig-like process branching off from shaft. From Jia and Gao, 2019 (character 71).
90. *Intermedium in the carpus: (0) smaller than or similar to the size of centrale; (1) larger than the centrale.
91. Fusion of distal carpal 1+2 into a single basale commune in the carpus (Shubin and Wake, 1996): (0) fusion absent; or (1) fusion present. From Jia and Gao, 2019 (character 72).
92. Fusion of distal tarsal 1+2 into a basale commune in the tarsus (Shubin and Wake, 1996): (0) fusion absent; (1) fusion present. From Jia and Gao, 2019 (character 73).
93. Number of centralia in manus and pes (Shubin and Wake, 1996): (0) more than one centralia; or (1) one or no centrale. From Jia and Gao, 2019 (character 74).
94. Distal tarsal 4 and 5: (0) separate; (1) fused. From Jia and Gao, 2019 (character 118).
95. *Metacarpal II: (0) expanded; or (1) not expanded.
96. *Metacarpal III: (0) expanded; or (1) not expanded.
97. Number of toes: (0) 4; or (1) 5. From Zhao and Hu, 1984 (character 14) and Jia and Gao, 2019 (character 120).
98. Claws or horny cover on digits or toes: (0) present; or (1) absent. From Zhao and Hu, 1984 and Jia and Gao, 2019 (character 27).

Soft tissue and developmental pattern

99. Lungs: (0) normally developed; or (1) reduced; or (2) entirely lost. From Jia and Gao, 2019 (character 98).
100. Pubotibialis and puboischiotibialis muscles: (0) separate; or (1) fused. From Duellman and Trueb, 1986 (character AA) and Jia and Gao, 2019 (character 89).
101. Pattern of vertebral development (Boisvert, 2009): (0) neural arch developed before centrum; (1) centrum developed before neural arch. From Jia and Gao, 2019 (character 100).
102. * Patterns of mesopodial ossification: (0) preaxial column ossifies later than the remaining mesopodials; (1) vice versa.

103. Haploid chromosome number (modified from Duellman and Trueb, 1986: character DD): (0) ranging from 20 to 32; (1) reduced to 19; (2) further reduced to 14 or less. From Jia and Gao, 2019 (character 77).

104. Diploid chromosome number (modified from Duellman and Trueb, 1986: character DD): (0) 56 or more; (1) 40-55; (2) lower than 40. From Jia and Gao, 2019 (character 78).

105. Microchromosome: (0) present; (1) absent. From Duellman and Trueb, 1986 (table 16-2) and Jia and Gao, 2019 (character 79).

106. Spermathecae in cloaca: (0) absent; or (1) present. From Sever, 1991 (character M) and Jia and Gao, 2019 (character 84).

107. Mode of fertilization: (0) external; or (1) internal. From Duellman and Trueb, 1986 (character CC) and Jia and Gao, 2019 (character 97).

108. *Mesopodium ossification: (0) absent; (1) present.

Data S1 Data matrix of 23 taxa by 108 characters used for cladistic analysis in this study, Related to Figures 6, S5 and STAR Methods

#NEXUS
[written Fri May 21 16:56:04 EDT 2021 by Mesquite version 3.04 (build 725) at LAPTOP-HJNQ13UA/192.168.1.164]

BEGIN TAXA;
TITLE Taxa;
DIMENSIONS NTAX=23;
TAXLABELS
Karaurus Chunerpeton Andrias_davidianus Cryptobranchus Pangerpeton Linglongtriton Neimengtriton Nuominerpeton Liaoxitriton Regalerpeton Onychodactylus_japonicus Paradactylodon_mustersi Ranodon Pachyhynobius Hynobius_amjiensis Salamandrella Batrachuperus_yenyuanensis Protohynobius Pseudohynobius_flavomaculatus Beiyanerpeton Qinglongtriton Liua_shihi Ambystoma_maculatum
;

END;

BEGIN CHARACTERS;
TITLE Character_Matrix;
DIMENSIONS NCHAR=108;
FORMAT DATATYPE = STANDARD GAP = - MISSING = ? SYMBOLS = " 0 1 2 3";
CHARSTATELABELS
1 dorsal_extension_of_alary_process_, 2 anterodorsal_fenestra, 3 anterodorsal_fenestra_border, 4 maxilla_size, 5 anterior_process_of_maxilla, 6 maxillary_tooth_row, 7 'maxilla/nasal contact', 8 'maxilla/frontal contact', 9 'maxilla/pterygoid bony contact', 10 septomaxilla, 11 lacrimal, 12 'lacrimal-narial-orbital border', 13 nasolacrimal_duct, 14 nasal_contact, 15 nasal_anterior_border, 16 lateral_expansion_of_nasal, 17 'nasal/prefrontal contact', 18 nasal_dimension, 19 lateral_wall_of_nasal_capsule, 20 lateral_narial_fenestra, 21 frontal_anterior_extension, 22 anterolateral_process_of_parietal, 23 'parietal/prefrontal contact', 24 position_of_the_trochlear_foramen, 25 frontoparietal_fontanelle, 26 size_of_fp_fontanelle, 27 'parietal/squamosal contact', 28 quadrate_distal_part_expansion, 29 quarate_nerve_foramen, 30 sculpture_of_skull_roof, 31 anteromedial_fenestra, 32 am_fenestra_size, 33 vomer_contact, 34 posterior_process_of_vomer, 35 'post-choanal process of vomer', 36 posterolateral_border_of_vomer, 37 vomerinet_teeth_location, 38 vomerine_tooth_row_direction, 39 'inner/outer vomerine teeth ', 40 length_of_cultriform_process, 41 cultriform_process_of_ps, 42 internal_carotid_foramen, 43 'parasphenoid/pterygoid contact', 44 palatine_in_adult, 45 palatine_tooth, 46

shape_of_pterygoid, 47 pterygoid_teeth, 48 anterolateral_process_of_pt, 49 length_of_anterolateral_process_of_pt, 50 direction_of_anterolateral_process_of_pt, 51 ossification_of_ascending_process, 52 orbitosphenoid_ossification, 53 anteroventral_extension_of_orbitosphenoid, 54 optic_foramen_, 55 oculomotor_foramen, 56 'prootic/opi/exo complex', 57 'otic-occipital complex midline contact', 58 operculumn, 59 ossified_stapes, 60 stapedial_foramen, 61 dentary_groove, 62 'angular/prearticular fusion', 63 coronoid_in_adult, 64 coronoid_teeth_in_adult, 65 articular_ossification, 66 marginal_teeth, 67 tooth_crown_of_marginal_teeth, 68 radial_loop, 69 paired_radial_loops, 70 'radial loop/ceratohyal contact', 71 cornua, 72 ceratohyal_ossification, 73 hypohyal_, 74 hypobranchial_I_and_ceratobranchial_I, 75 ossification_of_hypobranchial_I, 76 ceratobranchial_II_in_adults, 77 ceratobranchial_III_and_IV, 78 basibranchial_II, 79 basibranchial_II_shape, 80 tuberculum_interglenoideum_of_atlas, 81 transverse_process_of_atlas, 82 caudosacral_#, 83 atlantal_spinal_nerve_foramen, 84 head_of_postatlantal_ribs, 85 postatlantal_spinal_nerve_foramen, 86 'scapular/coracoid ratio', 87 coracoid_anteroposterior_extension, 88 dorsal_and_ventral_crests_of_humerus, 89 femoral_trochanter, 90 carpal_intermedium_size_with_centrala, 91 carpal_basale_commune, 92 tarsal_basale_commune, 93 number_of_centralia_in_manus_and_pes, 94 'dt 4+5', 95 metacarpal_II_expansion, 96 metacarpal_III_expansion, 97 toe_#, 98 claws_or_horny_cover_on_digits, 99 lungs, 100 'pubotibialis/puboischiotibitalias muscles', 101 vertebrate_development_pattern, 102 mesopodial_ossification_pattern, 103 haploid_chromosome_#, 104 Diploid_chromosome_#, 105 Microchromosome, 106 Spermathecae, 107 Mode_of_fertilization, 108 carpas_tarsals_ossifcation ;

MATRIX

Karaurus

00000?000?00?00010??000?0?00?001121121?000010011000??????000?0101??????0?001?0
01?000?1010?????111???????????0

Chunerpeton

000?00000?1??00110??010?0?00?101??01???000???0?1??001??0?0???0??0??????0?001?00
1011101000?????111???0???????0

Andrias_davidianus

00001011011?1001000011100?01?11?0001???0101101110000111100011010011 0??1011101
1?1021101000011{0 1}01111011?000000

Cryptobranchus

01?01001011?1001000011100?01?11?0001???0101101110000111100011010111 0??1111100
1?1021101000011{0 1}{0 1}1111011?000000

Pangerpeton

000000000?00?00010??000?0?00?1000?01????0021001100001100100??010011??????010?0
110111?1000?????111???????????0

Linglongtriton

00100000000000100 10??0000??00?100010220011021001100001020?0010010111????0??10?
0010011010 11?1111101????0?????1

Neimengtriton

0000001000010000 10??000?1100?100021220010?2100100000 1022?00000100??????0??10?
0010{0 1}11001110110 0111????0?????1

Nuominerpeton
000000100001?00010??00000?00010001021010102100110000102 1?0011010011????0??10?
0010{1 2}?10111101100011????0?????1
Liaoxitriton
000000?00????00010??000?0?00?1000112 10101?2100110000102 1?00100100??????0??00?0
110??1?111?0110?011????0?????1
Regalerpeton
00?000?00?03??0010??000?1100?101??01???0102??0?1??001021?00110???00????0?001?0
1101?1011110110?111????0?????1
Onychodactylus_japonicus 00{0 1}0000000030{1
2}1010000000 1111?10122122010102100 1100?000221001101001111 1200{0
1}1011?1021100111011 10111011?0000001
Paradactylodon_mustersi
00001010000100101000000001101?1002101202010210011101000221001101001 1???110000
1011021100111011{0 1}011000110000001
Ranodon
000010000000300101000000001011?100010110201021001110100022000{0
1}00100111112100101011021100111011000110011 0000001
Pachyhynobius
000011101002001010000000 00?01?1000201200010210011100000220001101001110111 0000
10110{1 2}1100111011001111011 0000001
Hynobius_amjiensis 000010{0
1}00003021010000000 0?01?100201220011021001110000021000010101 11???0011010110
211001110110001110110000001
Salamandrella
000010000000021010000000 01011?10120122001102100111000002 2000{0
1}1010011110100110101102110011101101010 1011 0000001
Batrachuperus_yenyuanensis 00001000000{0
3}0210100000000?010100010120201021001110{0
1}000210001101001110011011010110211001 11011{0 1}011000110000001
Protohynobius 000010000000300101000000001101?1010{1
2}0220001121001110000022000110101 11????00110101102110011101100011101 10000001
Pseudohynobius_flavomaculatus
00001000000302101000000001101010121122000112 1001110{0
1}00021000110101111011001101011 0{1 2}110011101100011101{0 1}0000001
Beiyanerpeton
001?10000003?10010??011?0?00?101??01???0002??0?1??00???0?00011??100????0?001?0
01?21021000????111??????????0
Qinglongtriton
001?1000000001001 0??010?0?00?101??01???0002??0?1??01???0?00001??110????0?001?0
11111031000????111??????????0
Liua_shihi 0000100000000{0
2}10100000000?01?10001011020102100 1110{0 1}00021000110100111111011010110{1
2}1100111011001110011 0000001

Ambystoma_maculatum
10201000001?0100101100000?110101121220000121001100000102011111101111???110?111
011121030011111101110010221111

;

END;
BEGIN ASSUMPTIONS;
TYPESET * UNTITLED = unord: 1 - 108;

END;

BEGIN MESQUITECHARMODELS;
ProbModelSet * UNTITLED = 'Mk1 (est.)': 1 - 108;
END;

Begin MESQUITE;
MESQUITESCRIPTVERSION 2;
TITLE AUTO;
tell ProjectCoordinator;
timeSaved 1621630564384;
getEmployee #mesquite.minimal.ManageTaxa.ManageTaxa;
tell It;
setID 0 8185193313307563739;
endTell;
getEmployee #mesquite.charMatrices.ManageCharacters.ManageCharacters;
tell It;
setID 0 5923896169336596133;
mqVersion 304;
checksumv 0 3 854495241 null getNumChars 108 numChars 108 getNumTaxa 23 numTaxa 23 short true bits 2305843009213693967 states 15 sumSquaresStatesOnly 6913.0 sumSquares -1.152921504606847E20 longCompressibleToShort false usingShortMatrix true NumFiles 1 NumMatrices 1;
mqVersion;
endTell;
getWindow;
tell It;
suppress;
setResourcesState false false 100;
setPopoutState 300;
setExplanationSize 0;
setAnnotationSize 0;
setFontIncAnnot 0;
setFontIncExp 0;

```
			setSize 766 736;
			setLocation 761 0;
			setFont SanSerif;
			setFontSize 10;
			getToolPalette;
			tell It;
			endTell;
			desuppress;
		endTell;
		getEmployee
#mesquite.charMatrices.BasicDataWindowCoord.BasicDataWindowCoord;
		tell It;
			showDataWindow					#5923896169336596133
#mesquite.charMatrices.BasicDataWindowMaker.BasicDataWindowMaker;
			tell It;
				getWindow;
				tell It;
					setExplanationSize 30;
					setAnnotationSize 20;
					setFontIncAnnot 0;
					setFontIncExp 0;
					setSize 666 664;
					setLocation 761 0;
					setFont SanSerif;
					setFontSize 10;
					getToolPalette;
					tell It;
						setTool
mesquite.charMatrices.BasicDataWindowMaker.BasicDataWindow.ibeam;
					endTell;
					setActive;
					setTool
mesquite.charMatrices.BasicDataWindowMaker.BasicDataWindow.ibeam;
					colorCells	#mesquite.charMatrices.NoColor.NoColor;
					colorRowNames
#mesquite.charMatrices.TaxonGroupColor.TaxonGroupColor;
					colorColumnNames
#mesquite.charMatrices.CharGroupColor.CharGroupColor;
					colorText	#mesquite.charMatrices.NoColor.NoColor;
					setBackground White;
					toggleShowNames on;
					toggleShowTaxonNames on;
					toggleTight off;
					toggleThinRows off;
```

```
		toggleShowChanges on;
		toggleSeparateLines off;
		toggleShowStates on;
		toggleAutoWCharNames on;
		toggleAutoTaxonNames off;
		toggleShowDefaultCharNames off;
		toggleConstrainCW on;
		toggleBirdsEye off;
		toggleShowPaleGrid off;
		toggleShowPaleCellColors off;
		toggleShowPaleExcluded off;
		togglePaleInapplicable on;
		toggleShowBoldCellText off;
		toggleAllowAutosize on;
		toggleColorsPanel off;
		toggleDiagonal on;
		setDiagonalHeight 80;
		toggleLinkedScrolling on;
		toggleScrollLinkedTables off;
	endTell;
	showWindow;
	getWindow;
	tell It;
		forceAutosize;
	endTell;
	getEmployee #mesquite.charMatrices.ColorByState.ColorByState;
	tell It;
		setStateLimit 9;
		toggleUniformMaximum on;
	endTell;
	getEmployee #mesquite.charMatrices.ColorCells.ColorCells;
	tell It;
		setColor Red;
		removeColor off;
	endTell;
	getEmployee #mesquite.categ.StateNamesStrip.StateNamesStrip;
	tell It;
		showStrip off;
	endTell;
	getEmployee #mesquite.charMatrices.AnnotPanel.AnnotPanel;
	tell It;
		togglePanel off;
	endTell;
	getEmployee
```

```
#mesquite.charMatrices.CharReferenceStrip.CharReferenceStrip;
				tell It;
					showStrip off;
				endTell;
				getEmployee #mesquite.charMatrices.QuickKeySelector.QuickKeySelector;
				tell It;
					autotabOff;
				endTell;
				getEmployee #mesquite.charMatrices.SelSummaryStrip.SelSummaryStrip;
				tell It;
					showStrip off;
				endTell;
				getEmployee
#mesquite.categ.SmallStateNamesEditor.SmallStateNamesEditor;
				tell It;
					panelOpen true;
				endTell;
			endTell;
		endTell;
		endTell;
end;
```
